# Amber in prehistoric Iberia: New data and a review

**DOI:** 10.1371/journal.pone.0202235

**Published:** 2018-08-29

**Authors:** Mercedes Murillo-Barroso, Enrique Peñalver, Primitiva Bueno, Rosa Barroso, Rodrigo de Balbín, Marcos Martinón-Torres

**Affiliations:** 1 Departamento de Prehistoria y Arqueología, Universidad de Granada, Granada, Spain; 2 Instituto Geológico y Minero de España (Geominer Museum), Valencia, Spain; 3 Departamento de Historia y Filosofía, Universidad de Alcalá de Henares, Madrid, Spain; 4 Department of Archaeology, University of Cambridge, Cambridge, United Kingdom; University at Buffalo - The State University of New York, UNITED STATES

## Abstract

Provenancing exotic raw materials and reconstructing the nature and routes of exchange is a major concern of prehistoric archaeology. Amber has long been recognised as a key commodity of prehistoric exchange networks in Europe. However, most science-based studies so far have been localised and based on few samples, hence making it difficult to observe broad geographic and chronological trends. This paper concentrates on the nature, distribution and circulation of amber in prehistoric Iberia. We present new standardised FTIR analyses of 22 archaeological and geological samples from a large number of contexts across Iberia, as well as a wide scale review of all the legacy data available. On the basis of a considerable body of data, we can confirm the use of local amber resources in the Northern area of the Iberian Peninsula from the Palaeolithic to the Bronze Age; we push back the arrival of Sicilian amber to at least the 4^th^ Millennium BC, and we trace the appearance of Baltic amber since the last quarter of the 2^nd^ Millennium BC, progressively replacing Sicilian simetite. Integrating these data with other bodies of archaeological information, we suggest that the arrival of Baltic amber was part of broader Mediterranean exchange networks, and not necessarily the result of direct trade with the North. From a methodological perspective, thanks to the analyses carried out on both the vitreous core and the weathered surfaces of objects made of Sicilian simetite, we define the characteristic FTIR bands that allow the identification of Sicilian amber even in highly deteriorated archaeological samples.

## Introduction

Amber and other unusual materials such as jade, obsidian and rock crystal, have attracted interest as raw materials for the manufacture of decorative items since Late Prehistory, and indeed amber retains a high value in present-day jewellery. There are many aspects that come into play when assessing the social value of these raw materials, ranging from intrinsic material properties (colour, texture, hardness …) that can render them appealing for the manufacture of adornments, to other environmental or cultural aspects that may relate, for example, to their relative scarcity and subsequent added value as exotic or 'exclusive'. As such, the circulation of unusual raw materials can be traced back to Prehistory, and the Iberian Peninsula is no exception.

For obsidian and jade, this movement is known to have taken place since early dates in the Late Prehistory. For instance, we can mention the Lipari and Pantelleria obsidian circuits in the Central Mediterranean that reached North Africa between the 6^th^ and the 2^nd^ Millennia BC [1–3]; the Alpine jade axes that circulated in Western Europe reaching Britain and the South-West of the Iberian Peninsula since at least the 5^th^ Millennium BC [4–6]; or the variscite from the South-West of the Iberian Peninsula that has been documented in French megaliths from the 4^th^ Millennium BC [7].

As for amber, there is evidence of use since the Late Palaeolithic (e.g. [8]). In a previous paper [9], two of us presented a diachronic perspective of the patterns of amber use in the Prehistory of the Iberian Peninsula, as well as of the sources that supplied it and the contact and exchange circuits that occurred as a result. In broad terms, we observed: the use of local resources in the Northern parts of the Peninsula (the area in which the largest geological deposits of amber are concentrated) from the Palaeolithic to the Bronze Age; a significant increase in amber items during the Neolithic and especially the Chalcolithic period across the whole Iberia, concurrent with the arrival of foreign amber–in particular Sicilian simetite; and a decrease in the use of amber during the Bronze Age, which appeared restricted geographically to the North-East of the Peninsula, when all of the analysed amber is of Baltic origin. Baltic amber again appeared with increasing frequency during the Late Bronze Age and Early Iron Age. However, this proposal was based on very limited archaeometric evidence, including only 18 archaeological sites with objects sampled, out of the over 85 where amber objects have been documented (Table 1).

**Table 1:** Number of archaeological amber objects and FTIR analyses available and the contribution of this paper.

Period	N. amber objects analysed / MNI amber objects in 2012 [9]	% Objects with FTIR analyses in 2012 [9]	N. amber objects analysed / MNI amber objects in 2018 [this paper]	% Objects with FTIR analyses in 2018 [this paper]
Palaeolithic/Mesolithic (35000-6^th^Mill. BC)	3 / 20	15.0%	3 / 20	15.0%
Neolithic (5^th^-4^th^Mill. BC)	3 / 78	3.8%	5 / 79	6.3%
Chalcolithic (3^rd^Mill. BC)	5 / 306	1.6%	48 / 309^a^	15.5%
Chalcolithic + Bronze Age (reused) (3^rd^-2^nd^Mill. BC)	0 / 28	0%	3 / 28	10.7%
Bronze Age (2^nd^Mill. BC)	7 / 183^b^	3.8%	7 / 185	3.8%
Late Bronze Age/Early Iron Age (Late 2^nd^–Early 1^st^Mill. BC)	7 / 548^c^	1.3%	8 / 548	1.5%
Total	25 / 1163	2.1%	74 / 1169	6.3%

^a^ This figure includes the 251 beads from the tholos of Montelirio, of which 35 samples analysed yielded virtually identical spectra.

^b^ This figure includes 135 beads found forming a single necklace in Muricecs.

^c^ The specific number of amber objects is not always stated. When ‘several amber beads’ are reported, we have quantified MNI = 2. When ‘X beads of glass and amber’ or ‘X beads of carnelian and amber’ is stated, we have quantified MNI = X amber beads, even if the actual number of amber beads must be smaller.

Here we present a new set of analyses of archaeological and geological amber that greatly expands previous coverage. Taking advantage of the growing body of data, and synthesising all the information available, this paper sets out to provide an up-to-date review of the evidence concerning the nature, distribution and circulation of amber in Prehistoric Iberia. This holistic perspective is contextualised more broadly in current discussion about the circulation of materials, knowledge and people in European Prehistory.

## Materials and methods

In the last five years, there have been major developments in our understanding of Iberian amber. From a geological point of view, we highlight the project 'Iberian Amber: An Exceptional Record of Cretaceous Forests at the Rise of Modern Terrestrial Ecosystems', with the involvement of one of us (E.P.), focused on the palaeoenvironmental reconstruction of the ecosystems that generated the large Cretaceous amber deposits known in the Iberian Peninsula (e.g. [10]). From an archaeological point of view, the publication of the monograph on the tholos of Montelirio, in Valencina de la Concepción, Seville [11]–a large Megalithic monument were more than 250 amber objects were deposited among other exotic materials–has revealed the largest assemblage of amber objects from Iberian Prehistory to date [12]. Furthermore, we have had opportunity to analyse amber from some of the most iconic sites in the South of the Iberian Peninsula, such as the tholos of Montelirio and the Dolmen of La Pastora in Valencina de la Concepción, or the site of Los Millares. Two sites further inland and also sampled, the megalithic tomb of La Velilla and the hypogeum of the Valle de las Higueras, have provided data to complement that from the hypogeum of Almada. These new analyses increase our coverage of Chalcolithic sites with amber from 1% to 15% (Table 1), thus allowing a rather more representative view than possible in 2012. Equally, the new analyses from Quinta do Marcelo, Llano de la Teja 18 and Llano de la Sabina 97 and 99 constitute the first analytical data for these very well-known contexts and they open up interesting opportunities. In addition to archaeological samples, we have expanded the characterisation of geological samples from six areas of the Iberian Peninsula, following identical protocols and hence ensuring data compatibility. These include deposits of amber from the Cantabrian Range, Navarra, Asturias, Teruel, Castellón and Jaén (Table 2). (See S1 File).

**Table 2:** Specimens analysed in this work. No permits were required for the analysis of geological samples. Permits for the analyses of archaeological samples were granted by the Chief Curators and Heads of the museums listed in the table, under the R&D projects: HAR2017-82685-R, funded by the Spanish Ministry of Science, Innovation and Universities; and PN623183 funded by the European Commission.

ID	Geological locality (G) / Archaeological site (A)	Province	Museum
Ajo_340_1	Ajo (G)	Cantabria	Geominer Museum. Madrid
Comillas_412_1	Comillas (G)	Cantabria	Geominer Museum. Madrid
Cuchía_334_1	Cuchía (G)	Cantabria	Geominer Museum. Madrid
Arrudo_333_1	Puente ‘El Arrudo’ (G)	Cantabria	Geominer Museum. Madrid
Alloz_337_1	Alloz (G)	Navarra	Geominer Museum. Madrid
Zubieki_411_1	Zubielki (G)	Navarra	Geominer Museum. Madrid
Caleyu_341_1	El Caleyu (G)	Asturias	Geominer Museum. Madrid
S_Just_336_1	Sant Just (G)	Teruel	Geominer Museum. Madrid
Arenoso_410_1	La Hoya (G)	Castellón	Geominer Museum. Madrid
Navalperal_335_1	Navalperal (G)	Jaén	Geominer Museum. Madrid
360	La Velilla (A)	Palencia	Provincial Archaeological Museum. Palencia
38557	Dolmen of La Pastora (A)	Sevilla	National Archaeological Museum. Madrid
1976/1/MILL/12/2	Los Millares (A)	Almería	National Archaeological Museum. Madrid
1976/1/MILL/63/1	Los Millares (A)	Almería	National Archaeological Museum. Madrid
76/1/MILL/74/24	Los Millares (A)	Almería	National Archaeological Museum. Madrid
1985/49/SABINA/99/62	Llano de la Sabina (A)	Granada	National Archaeological Museum. Madrid
SABINA/97/2	Llano de la Sabina (A)	Granada	National Archaeological Museum. Madrid
LLTEJA/18/4	Llano de la Teja (A)	Granada	National Archaeological Museum. Madrid
VH/C1/C1/II/11	Valle de las Higueras (A)	Toledo	Santa Cruz Museum. Toledo
MAH7771SPII1330	Sao Paulo (A)	Almada	Municipal Museum of Almada
MAH10503SPII1521	Sao Paulo (A)	Almada	Municipal Museum of Almada
MAH1745QMar354	Quinta do Marcelo (A)	Almada	Municipal Museum of Almada

In order to characterise the samples, analyses were carried out via Fourier Transform Infrared Spectroscopy (FTIR), which is well-established as a reliable technique for the characterisation and sourcing of amber. The spectra obtained were compared with both reference spectra of geological samples previously available, and the new geological spectra presented in this article. The new specimens were analysed using a Perkin Elmer Spectrum Two FTIR spectrometer at the Wolfson Archaeological Science Laboratories of the UCL Institute of Archaeology (UK). This instrument allows for Attenuated Total Reflection (ATR) analyses and hence potentially totally non-invasive analyses. However, most of the archaeological objects studied here are held or exhibited at various museums in Spain and Portugal, and for curatorial concerns the extraction of a very small sample was preferable to moving the whole objects. Therefore, pellets were made using approximately 2 mg of analyte which was ground by hand in an agate mortar and mixed with a small amount of KBr, before pressing the mixture in a 13 mm diameter mould in order to produce 1 mm thick discs. The data were collected as infrared transmission spectra after scanning each specimen 50 times in the range 4000–370 cm^-1^, with a resolution of 4 cm^-1^. All of our raw spectra are included in full as Supporting Information (S1 File) to facilitate future reuse.

### Amber resources in Iberia

**Fig 1:**
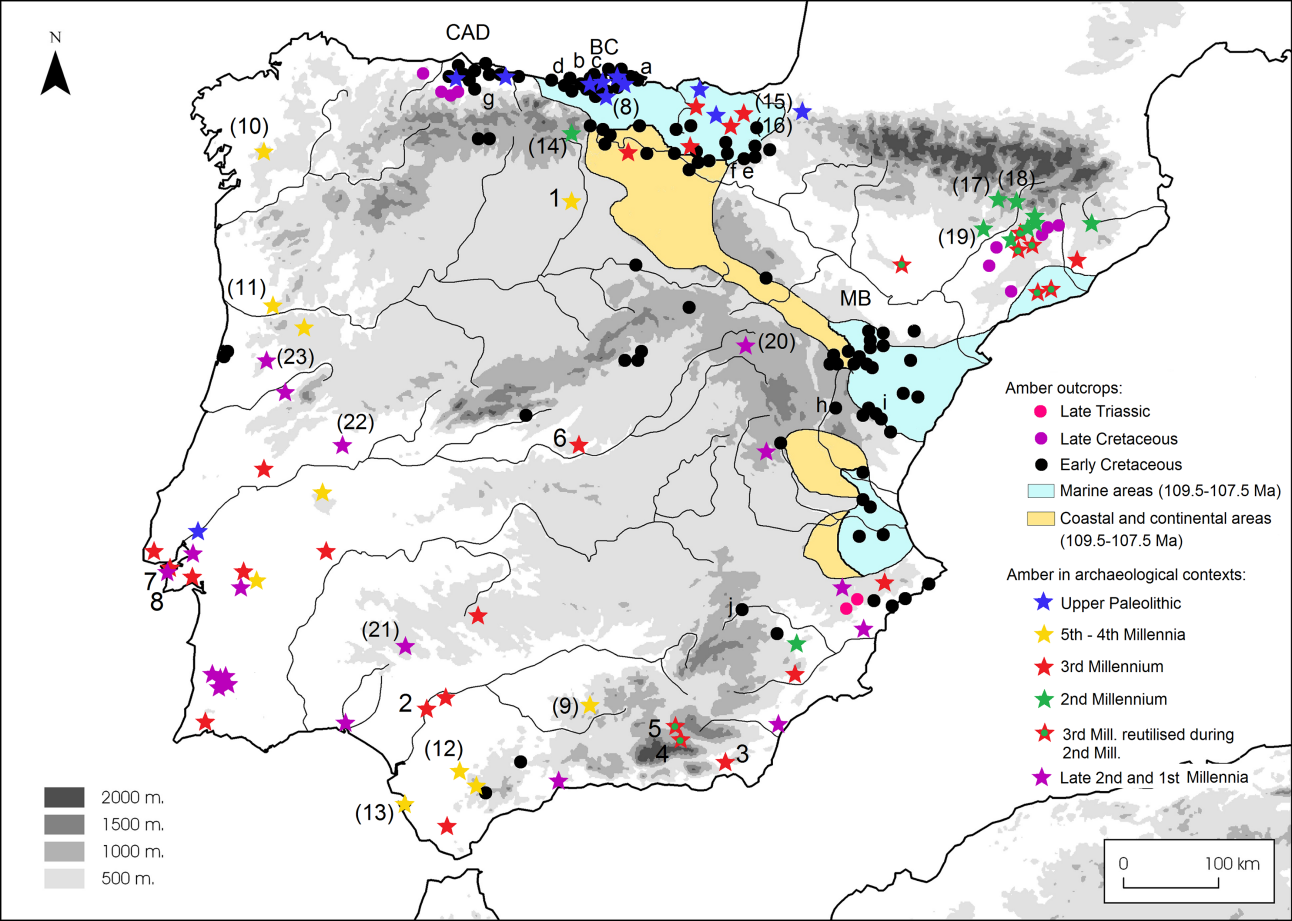
Location of geological (dots) and archaeological (stars) amber findings. Amber outcrops in Iberia modified from [10] and archaeological findings modified from [9]. CAD: Central Asturian Depression; BC: Basque-Cantabrian Basin; MB: Maestrazgo Basin. Geological samples analysed in this paper from: a) Ajo; b) Comillas; c) Cuchía; d) Puente ‘El Arrudo’; e) Alloz; f) Zubielki; g) El Caleyu; h) Sant Just; i) La Hoya and j) Navalperal. Archaeological objects analysed in this paper from: 1. La Velilla; 2. Valencina de la Concepción; 3. Los Millares; 4. Llano de la Sabina (97 and 99); 5. Llano de la Teja 18; 6. Valle de las Higueras; 7. Artificial cave of Sao Paulo and 8. Quinta do Marcelo. Other archaeological sites mentioned in this paper in order of appearance in the text: (8) La Garma A; (9) Los Cuarenta Cave; (10) Chousa Nova; (11) Dolmen de Mamoa V de Chã de Arcas; (12) Dolmen de Alberite; (13) Campo de Hockey; (14) Los Lagos I; (15) Larrarte; (16) Trikuaizti I; (17) Pedra Cabana; (18) Cabana del Moro de Colomera; (19) Muricecs; (20) Herrerías II; (21) Palacio III; (22) Moreirinha and (23) Senhora da Guia.

Around 150 localities with Cretaceous amber have been documented in the Iberian Peninsula [10, 13]. Most of these amber deposits are Albian in age (Early Cretaceous) and only some localities in Asturias and in Catalonia date from the Late Cretaceous. Likewise, only two localities with amber from the Late Triassic Period are known, both in Alicante. In general, the amber deposits are distributed in a strip that goes from the East to the North of the Iberian Peninsula, corresponding approximately with the marine coastline during the Early Cretaceous (Fig 1). In this strip, amber deposits are mainly concentrated in three geological contexts: The Maestrazgo Basin, the Basque-Cantabrian Basin and the Central Asturian Depression [10, 14]. Towards the South, three localities with relatively minor amber occurrences are known in Puerto del Boyar (Cádiz) [15, 16]; Navalperal (Jaén) and Almargen (Málaga). To this day, two of these three localities lack systematic study and FTIR characterisation, and in the last case of Almargen, the references are vague: in the early 20^th^century Mr. S. Calderón [17] referred to the existence of a unique piece of amber from Almargen in the possession of Mr. F. Chaves [18].

Some of the amber deposits have undergone intense palaeobotanic and geochemical study. There have been systematic studies using FTIR of the most important localities such as Sant Just in the Maestrazgo Basin, or Peñacerrada and El Soplao in the Basque-Cantabrian Basin [19–22], to which we can add analyses of individual samples from Cóbreces and Comillas in the Basque-Cantabrian Basin; La Clusa in Catalonia; Hoz Seca in Guadalajara or the aforementioned analysis of Puerto del Boyar [8, 10, 16, 23, 24].

**Fig 2:**
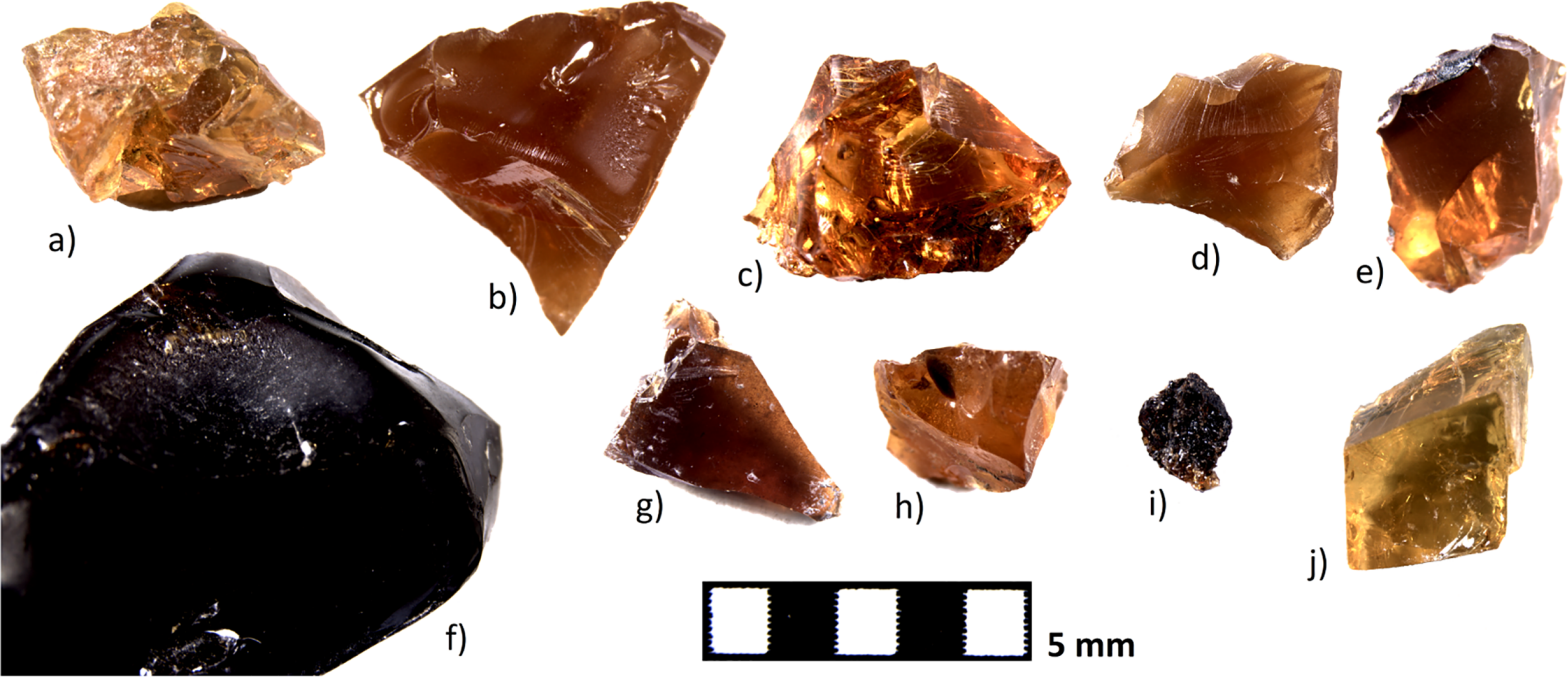
Geological amber samples analysed. a) Ajo, b) Comillas, c) Cuchía, d) Puente ‘El Arrudo’, e) Alloz, f) La Hoya, g) San Just, h) El Caleyu, i) Navalperal, j) Zubileki.

Geological samples of the main deposits have been selected as references in the present work. One sample from Jaén province (Navalperal) has also been included due to its closer proximity to some of the archaeological sites considered. The geological samples analysed are presented in the following sections (Fig 2).

#### Basque-Cantabrian Basin (Localities of Ajo, Comillas, Cuchía and Puente El Arrudo in Cantabria, as well as Alloz and Zubielki in Navarra)

There is a large sedimentary basin that developed during the Cretaceous period between the Iberian and European tectonic plates. The main deposits of amber in this area are those of El Soplao and Peñacerrada [19, 21], which both belong to the Utrillas Group [14].

Amber is documented in clayey rocks rich in organic matter from sediments deposited mainly in coastal areas and fluvial channels. There is a large quantity of plant remains, coal and other continental organic material transported by rivers. Some deposits contain remains of marine life as molluscs, which indicate a coastal environment in the area in which the resin was buried [21].

The FTIR spectra for amber from this area had been previously determined through the study of the Peñacerrada and El Soplao deposits in particular [19, 21], and they are characterised by small C-H bands around 2950 cm^-1^, bands at 1460 and 1380 cm^-1^ because of simple C-H bonds, and bands from the group of carbonyls around 1700 cm^-1^, with the absence of bands at 3070 cm^-1^ being consistent with the amber's high degree of maturity.

**Fig 3:**
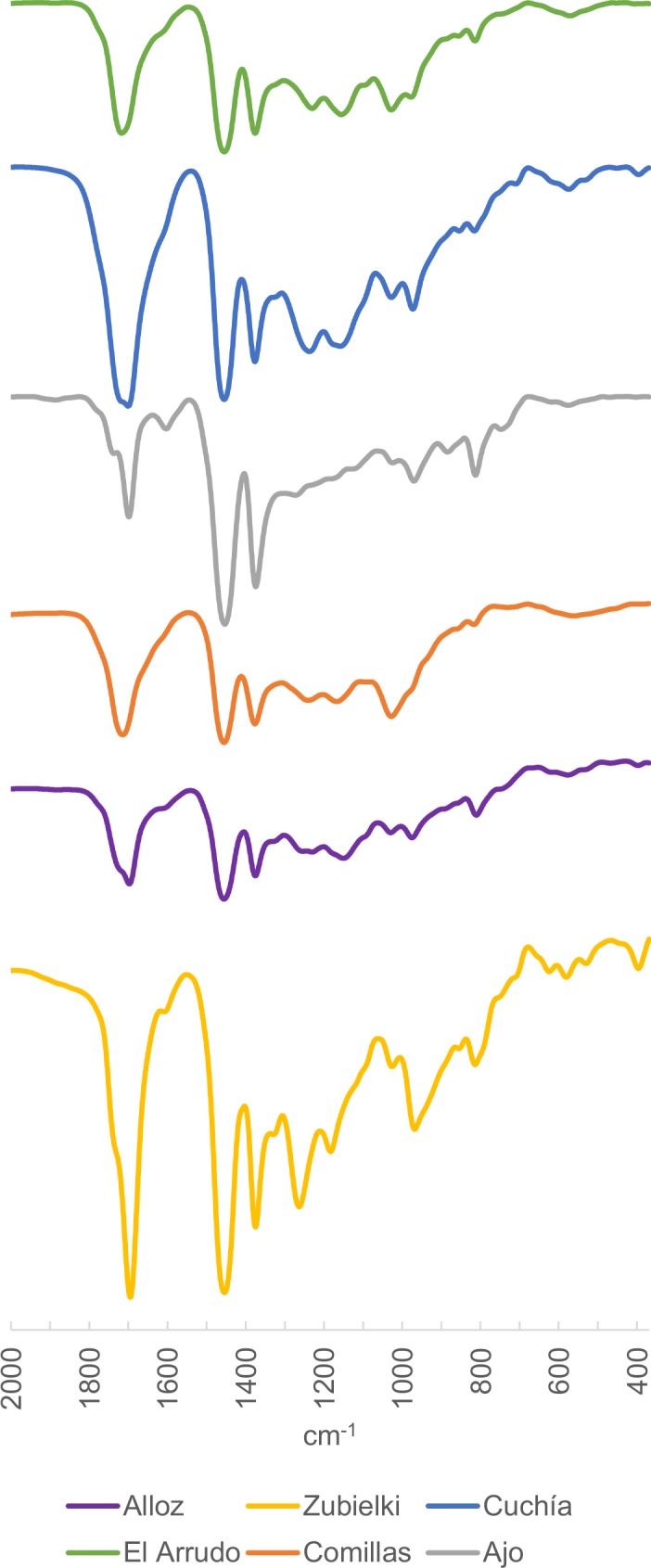
FTIR Spectra of palaeontological amber from the Basque-Cantabrian Basin.

We selected amber samples from six other localities (Ajo, Comillas, Cuchía and Puente El Arrudo in Cantabria, as well as Alloz and Zubielki in Navarra) to explore variability across the entire area. In general, the spectra have the same characteristics as those already published for El Soplao and Peñacerrada, with the sample from Ajo presenting a spectrum that is slightly different from the rest (Fig 3): all the spectra show the aforementioned bands at 1456 and 1375 ±5 cm^-1^, a band with greater or lesser intensity at 1270 ±5 cm^-1^ preceded sometimes by a secondary peak and two secondary bands at 1180 and 1025 ±5 cm^-1^ followed in most instances by a band at 970 ±5 cm^-1^.

#### Central Asturian Depression (locality of El Caleyu)

In the Central Asturian Depression, the main known amber deposits are those documented in the El Caleyu and Ullaga formations. In Pola de Siero, amber occurs, along with pyrite and fossilised plants, in lenses of grey silt within a 50-metre-deep layer of siliceous conglomerates, with a silt-sand matrix deposited on the Palaeozoic levels [25]. In the Southern area of Pola de Siero, in the Ullaga Formation (defined from deposits in the hill of Ullaga), amber is also documented in levels of grey silt with abundant lignite, oysters, ostracods and selachians. In the amber pieces, which can reach 20 cm in length, insects and other arthropods can be identified as bioinclusions [26, 27]. The locality of El Caleyu belongs to the Ullaga Formation. In the El Caleyu Formation, amber appears in a similar form, next to pyrite and accumulations of allochthonous lignite, in lenses of black silt within a 25-metre-thick layer of weakly cemented white or yellowy-white sandstones [25–27]. Amber also occasionally appears in other formations [10], for instance in the La Manjoya Formation, which is lithologically very similar to the Ullaga Formation [26, 27].

**Fig 4:**
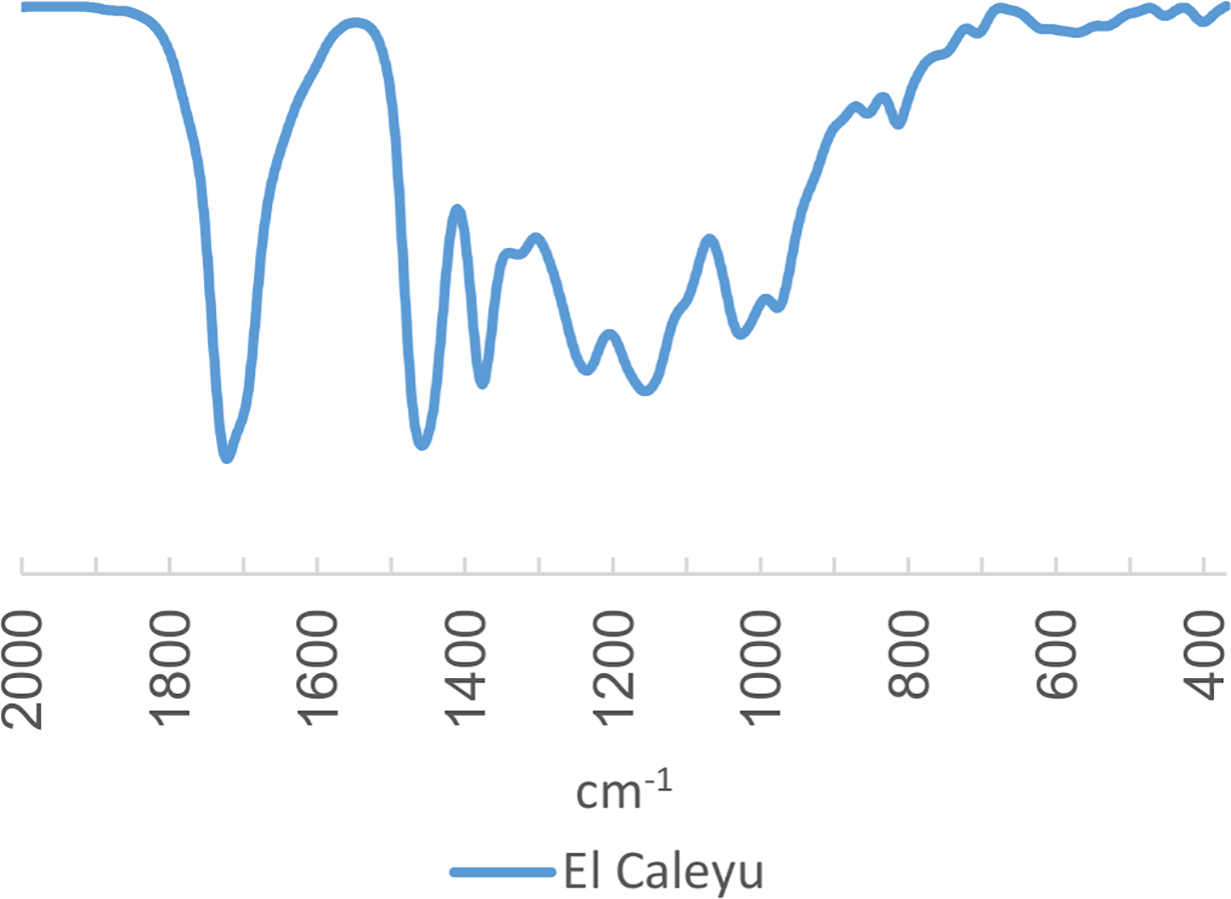
FTIR spectrum of palaeontological amber from El Caleyu (Central Asturian Depression).

There are some studies of the bioinclusions of these amber deposits, as well as gemmological studies, although prior to this work no FTIR spectra have been published. For the present research, we selected samples of amber from El Caleyu, whose FTIR spectra were similar to those already published for other Cretaceous Peninsular amber (Fig 4).

### The Maestrazgo Basin (Localities of San Just, Teruel, and La Hoya in Castellón)

The Maestrazgo Basin, which reached a sediment thickness of 6.5 km during the Mesozoic, is an intra-continental basin located in the Iberian Range that underwent two significant stages of rift during the Late Jurassic and Early Cretaceous; the rift structure is characterised by a system of faults that divide the Maestrazgo Basin into different blocks [28]. In general, sedimentation is dominated by sandstones, limestones and marlstones deposited in a marine environment with shallow freshwater environments. Amber is generally associated with organic silt deposits containing coal [10], with the largest deposits being San Just, La Hoya and Arroyo de la Pascueta, all in the Escucha Formation [10] or perhaps the Utrillas Group, according to the new data published by Barrón et al. [14] from the amber deposits in the Basque-Cantabrian Basin.

The San Just deposit has grey-black clay stones with abundant plant remains and fusinised wood. Palynological studies suggest a subtropical hot-humid environment with hot and dry areas [22].

The FTIR spectrum from San Just was published by Peñalver et al. [22] and is characterised, just like the spectrum from Álava [19], by two intense bands at 1724 cm^-1^ corresponding to the carbonyl group and the simple C-H bond respectively, with another band at 856 cm^-1^. The absence of the band of the exocyclic methylene group at 1640 and 880 cm^-1^ is significant, as it indicates a high level of maturity (including diagenetic effects) which would be consistent with its Cretaceous age.

**Fig 5:**
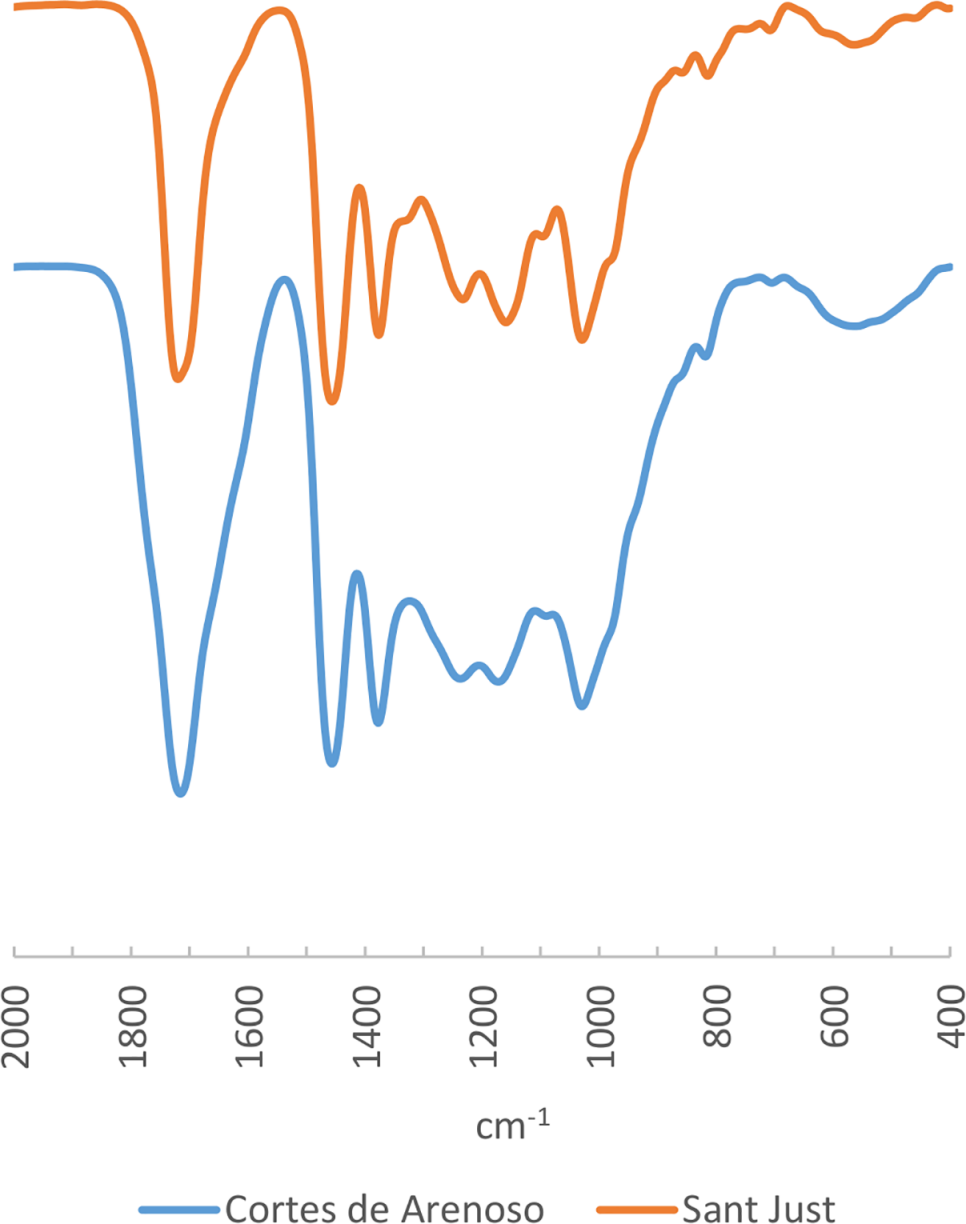
FTIR Spectra of palaeontological amber from the Maestrazgo Basin.

The La Hoya deposit, in Cortes de Arenoso municipality, contains abundant amber combined with coals and other vegetal remains deposited in deltaic environments. Insects have been documented in the amber as bioinclusions [10]. The samples from these localities analysed by us present practically identical spectra in which we observe the same general characteristics as in the previous samples (Fig 5).

#### Jaén (locality of Navalperal)

In a stratigraphic cross-section of Navalperal (Jaén), near the town of Orcera, in the Prebetic Domain, a few pieces of amber have also been identified, a few centimetres in diameter, in a level of dark limestone with lignite from the Ci3 sequence (see Fig 4.1 in [29]); according to these authors, the Ci3 sequence corresponds to a brief period with a scarce sedimentary record. This amber has shades ranging from yellow and orange to dark red or very dark, and dates from the Hauterivian-Barremian.

**Fig 6:**
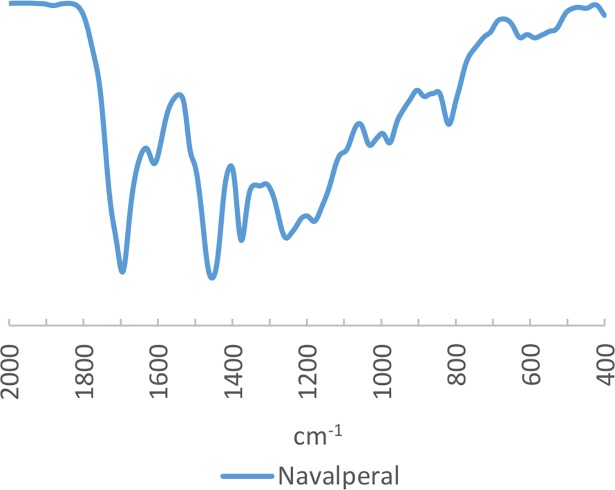
FTIR Spectra of palaeontological amber from Navalperal (Jaén).

To this day, such discoveries of amber in small quantities are unheard of. During the present research, we carried out FTIR analysis of this locality given its greater proximity to many of the archaeological sites studied, compared to the geological amber deposits previously discussed. However, the FTIR spectrum obtained do not differ qualitatively from those already known for the geological deposits of the Northern half of the Iberian Peninsula (Fig 6) and have the same characteristic bands in the diagnostic regions.

All in all, therefore, any of these spectra may be taken as characteristic reference spectra of Cretaceous Peninsular amber.

### Context of archaeological samples

The following sections provide some archaeological background to the samples reported here, while our discussion will integrate these new data with results published previously. Further details of archaeological samples analysed previously, and relevant bibliographic references, can be found in our previous publication [9].

#### La Velilla (Osorno, Palencia)

The burial monument of La Velilla is a circular chamber of about 9.5 m in diameter in which a minimum of 71 individuals were buried (56 adults and 15 sub-adults) of which only 9 retained anatomical connection [30]. Five amber beads were recovered among the grave goods along with a series of beads and pendants (55 in total) of other materials including lignite or variscite, as well as idols in bone and 22 pieces of unmanufactured raw materials such as deer antler, quartz, bone, or teeth [30, 31]. Most of these materials cannot be associated directly with any individual, except for an amber bead which, together with five lignite beads, appears to be directly associated with one of the skulls in the form of a necklace [30, 31].

**Fig 7:**
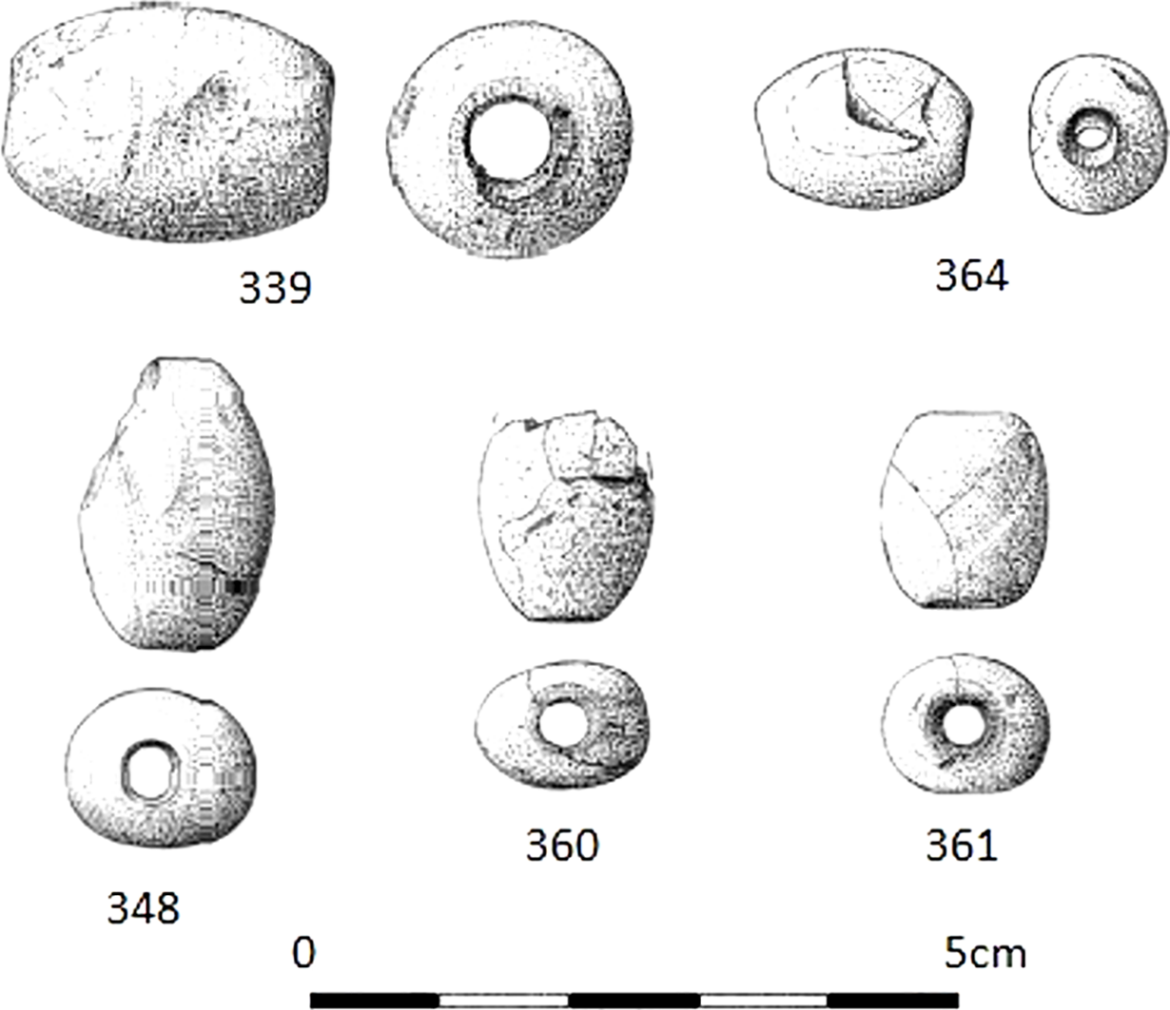
Drawing of amber beads from La Velilla. Numbers correspond to inventory. The bead analysed for this study is number 360. Drawing modified from [30].

The five amber beads have a similar barrel shape, ranging between 1.6 and 2.5 cm in length and with a maximum diameter of 1.2–1.9 cm (Fig 7). We analysed bead 360.

A recent series of 13 radiocarbon dates (one on charcoal, one on animal bone and 11 of buried individuals) place the use of this monument between the second half of the 4^th^ Millennium and the first half of the 3^rd^ Millennium BC (3635–3374 and 3011–2877 cal. BC) [30].

#### Valencina de la Concepción (Sevilla)

The site of Valencina de la Concepción (c. 3200–2300 cal. BC) holds the greatest number of amber objects known for a single site for all the Late Prehistory of the Iberian Peninsula, and it stands out both for its size and for the uniqueness of some of its graves and materials (e.g. [11, 32, 33]; for detailed discussion of its chronology see [34]). In Valencina de la Concepción, 254 objects of amber have been identified, of which 251 come from the tholos of Montelirio, two from the nearby grave 10042–10049 of PP4 Montelirio, and one last piece from the Dolmen of La Pastora.

**Fig 8:**
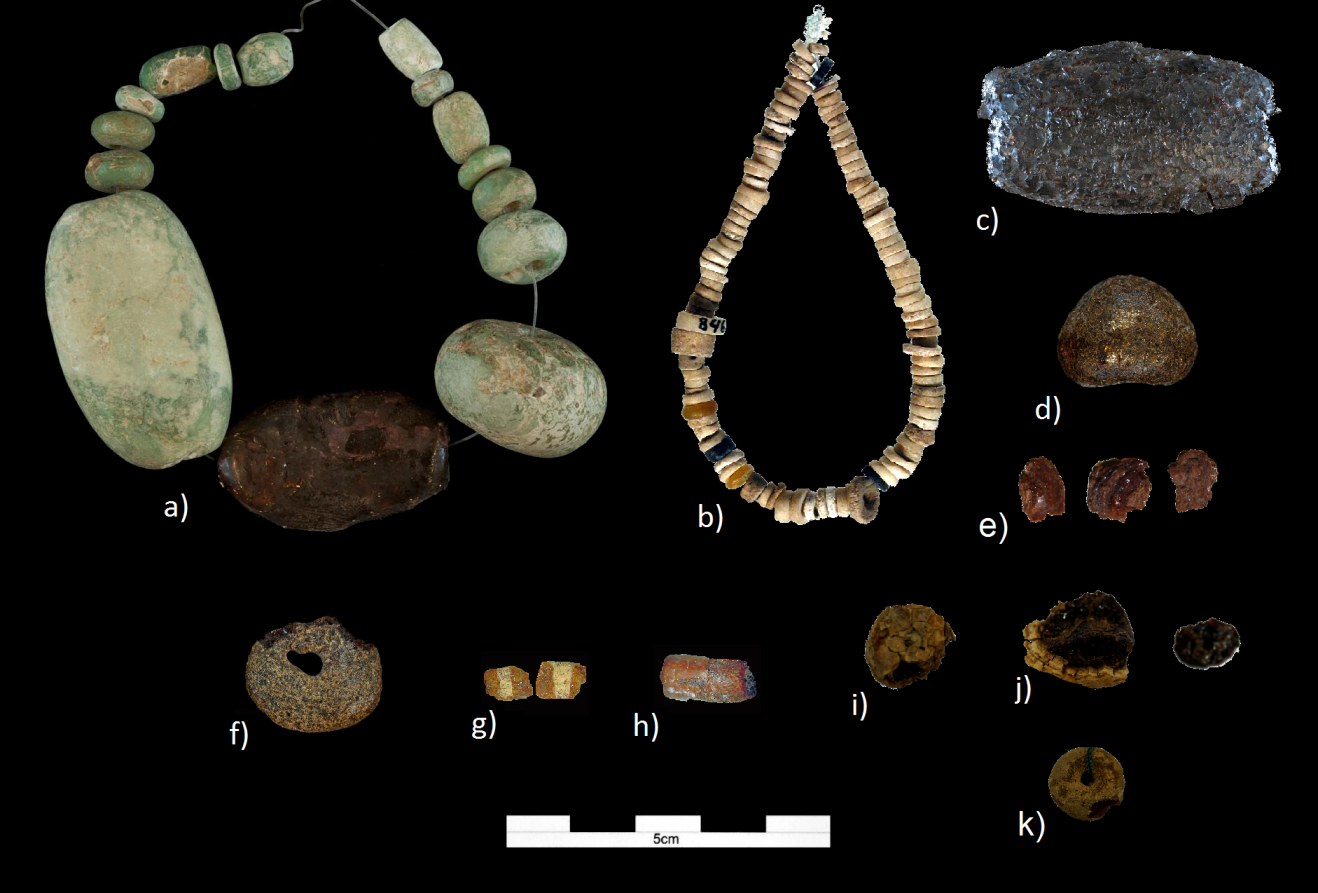
Some of the amber objects analysed. a) Bead from Dolmen of La Pastora (Valencina de la Concepción); b) Beads recorded as amber from Los Millares 7(VII); c) Bead from Los Millares 12; d) Amber objects from Los Millares 63(III); e) Amber bead from Llano de la Sabina 99; f) Bead from Valle de las Higueras 1; g) Bead from Valle de las Higueras 3; h) Bead from Sao Paulo MAH7771_SPII1330; i) Bead from Sao Paulo MAH10503_SPII1521; j) Bead from Quinta do Marcelo MAH1745_QMar354.

**Fig 9:**
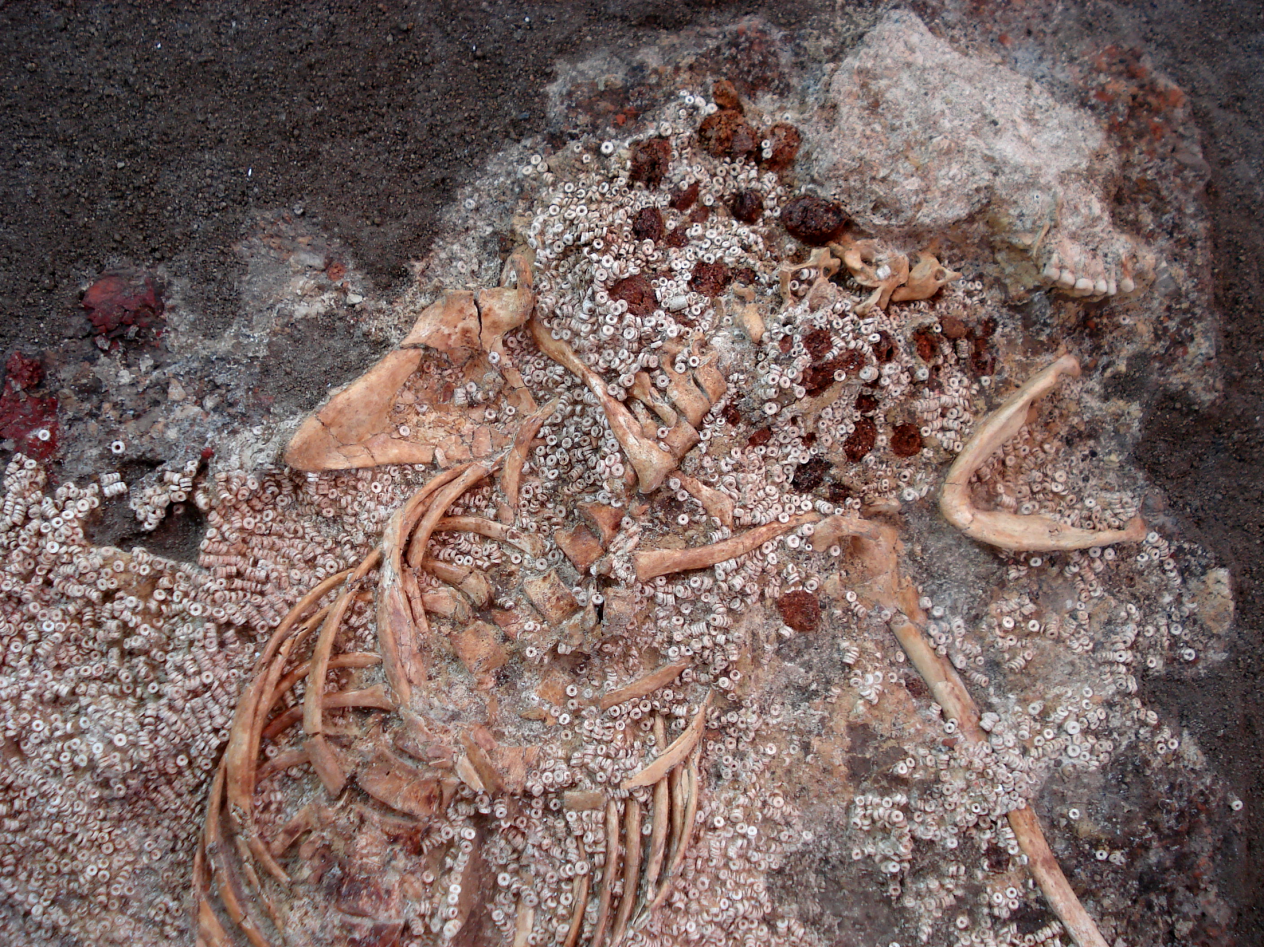
Example of one of the dresses made of amber and shell beads from the tholos of Montelirio. From [12].

- The Dolmen of La Pastora (c. 2500 cal. BC). The amber object from this monument is a large bead, 3.3 cm in length and 0.9 cm wide, currently on permanent display at the National Archaeological Museum (Madrid) together with 14 green beads (Fig 8A). Until the recent excavations of the Montelirio area, this was the only object of amber known from the site. The Dolmen of La Pastora is a tholos with a corridor of 32 m in length, divided in three sections, which leads to a circular chamber of 2.5 m in diameter. It was discovered in 1860 and regrettably nothing exact is known about the position or associations of the human remains or offerings found at this site. As for the amber bead, we know that it was donated on July 3, 1945 to the Museum by the Duchess Viuda de la Unión de Cuba and the Countess of Peña Ramiro, and that previously, it had belonged to the Count of Castilleja de Guzmán [35]. The set of objects donated, as recorded in the Museum's entry record, is made up of 11 flint arrowheads, a fragment of bone, a rectangular plate with two perforations, two sheets of gold, three copper sheets or plates and four copper punches, a fibula, two glassy pieces in the shape of truncated cones, six fangs, a tooth and a molar of a wild boar, 15 shells plus a necklace consisting of 166 shells, a reddish earthy fragment, two fragments of shiny reddish stones and a bracelet or necklace fragment composed of 14 green beads and a blackish one made of amber (Fig 8A).
- The tholos of Montelirio (3200–3100 cal. BC). This is a huge megalithic structure with a large corridor, 39 m in length and only 1.3 m high, which forces visitors to access the chamber by crawling. At the end of the corridor there is a chamber with a 4.75 m diameter and a second smaller chamber, 2.7 m in diameter. In total, 19 middle-aged women have been documented in the large chamber, recovered *in situ* around a cinnabar-covered stele, and a further two individuals in the small chamber whose sex could not be identified. All the women in the large chamber were dressed with costumes made up of more than a million beads of shell (and 251 of amber), covered with red cinnabar and accompanied by impressive grave goods consisting of gold-embossed sheets, carved ivory objects, ostrich eggs, large flint sheets and 2–4 mm thick flint arrowheads, rock crystal and amber. Similarly, the orthostats of the tholos were lavishly decorated in shades of red and black on their inner surface [36]. Most of the 251 amber objects were part of the women's clothing (Fig 9), although some possible unperforated figurines were also documented and could have been deposited as part of the grave goods [12] (for a detailed study of the tholos see [11]).
- Grave 10042–10049 (3515–2875 cal. BC). About 200 m North of the tholos of Montelirio is an area called PP4 Montelirio where more than 130 negative structures (megalithic and non-megalithic) have been documented, of which 61 contained human remains and 73 did not (see [37] for a summary of the results of this intervention). Of these structures, number 10042–10049 stands out [38], slightly earlier than the tholos of Montelirio, with a date of 3200–3100 cal. BC [34], and consisting of a corridor of 12 m in length, a first chamber of 2.6 m in diameter, followed by a second section of corridor 2.5 m long, which gives access to a second chamber of 2.2 m in diameter. In this second chamber an individual was found buried in anatomical connection and right lateral decubitus foetal position, covered with cinnabar, with an unworked elephant tusk framing the head, as well as a dish equally covered by cinnabar, 23 flint sheets, numerous ivory objects (many of them decorated) and a copper punch, as well as a flint halberd next to which was found what could potentially be the most unique piece of amber in the Recent Prehistory of the Iberian Peninsula: an amber pommel [9, 39] as well as small fragments of amber that appeared embedded in an ivory cylinder together with the larger piece.

Detailed studies of the amber objects from the tholos of Montelirio and of the pommel from the 10042–10049 structure, including FTIR analyses, have been reported elsewhere [12, 9, 39]. However, we will use the results in this article for comparative purposes.

#### Los Millares (Santa Fe de Mondújar, Almería)

Amber beads were recovered in the grave goods of several burial mounds at the Chalcolithic settlement of Los Millares. The materials collected during the excavations by P. Flores and L. Siret were deposited at the National Archaeological Museum and subsequently published by V. and H. Leisner [40], while the materials from the excavations by M. Almagro and A. Arribas [41] were deposited in the Provincial Archaeological Museum of Almería, Spain. Specifically, amber objects were only reported in the burial mounds 7(VII), 8(IV), 12, 63(III) and 74(XIII) (the Arabic numbering is that given by the Leisners, and the renumbering in Roman Numerals was given by A. Almagro and A. Arribas in their review of the material).

- Mound 7(VII). This is a tholos with a circular chamber that is 4.3 m in diameter and with a 3 m long corridor where the remains of 50 individuals were documented. Amongst other grave goods, three amber beads were recovered [40], although there is no greater description or illustrations of them. According to the review carried out by Almagro and Arribas [41] only shapeless fragments with a maximum length of 1 mm were left at the National Archaeological Museum, and they were in a very poor state of preservation. In addition, two discoidal beads 0.8 cm in diameter and displayed at the Provincial Archaeological Museum of Almería alongside a set of stone beads are catalogued as made from amber and coming from this tomb (Fig 8B). However, in our view, these seem too well preserved to constitute amber, and are more likely to be made of stone.
- Mound 8(IV). This is a mound with a trapezoidal chamber and a 2.9 m long corridor with burned remains of 20 individuals. The Leisners [40] do not mention the presence of any pieces of amber, although in the re-excavation of the tomb by Almagro and Arribas [41] a small and amorphous fragment of amber was documented and deposited in the Provincial Archaeological Museum of Almería.
- Mound 12. This is a tholos with a 3.8–4 m diameter chamber and a 2.4 m long corridor in which 12 individuals and five pieces of amber were recovered: a fragment of a cylindrical bead; a drop or tear drop-shaped bead with a "V" perforation; a smaller cylindrical bead with reddish pigmentation; a disc with an arched surface, and a barrel bead [40]. Regrettably, only this last bead has been preserved. It has a maximum length of 4.5 cm and a maximum width of 2.1 cm (Fig 8C) and was donated to the National Archaeological Museum by L. Siret in 1934. In the description made by the Leisners [40], they mention a series of longitudinal lines on its surface. These lines also appear represented in Siret's drawing [42]; however, these features cannot be distinguished today, as the bead currently shows a crackled black surface and obvious signs of degradation, seemingly the result of exposure to high temperatures.
- Mound 63(III). Architecturally, Mound 63(III) is a 4 m long mound with a trapezoidal chamber and a 3.5 m long corridor in which several individuals were documented [40]. Here a piece of rounded amber, with a flattened base, about 2 cm in diameter, was recovered. In this case, the artefact is not a bead because it has no perforation. It was donated to the National Archaeological Museum by L. Siret in 1934 and is currently exhibited at the Museum's permanent exhibition (Fig 8D).
- Mound 74(XIII). This is a tholos with a chamber 1.6 m high and a diameter of 4 m; the corridor has not been preserved [40]. Amongst the grave goods, a piece of amber is mentioned [40], but no precise description is given or shown in the album. Neither do Almagro and Arribas [41] mention any pieces of amber in their review of the tomb materials. Nowadays, three fragments are preserved at the National Archaeological Museum (Fig 8E). They exhibit a thick superficial weathered layer although the interior remains of vitreous appearance. Samples were taken both from the core and the surface layer.

#### Llano de la Sabina (97 and 99) (Gorafe, Granada)

At the set of graves at Llano de la Sabina, the appearance of amber beads is reported for two of them: graves 97 and 99.

- Llano de la Sabina 97 is a mound with a 2 x 1.7 m quadrangular chamber in which 'remains' of a skeleton were documented [40]. A circular or oval bead in an advanced state of degradation was also recovered, now held at the National Archaeological Museum.
- Llano de la Sabina 99 is a mound with a 2.7 m long trapezoidal chamber and a 4 m long corridor in which up to 20 individuals could have been buried [40]. Inside, a circular amber disk was found. It is about 2.2 cm in diameter, with a central perforation, and has two notches in one of its outer edges, although its state of conservation is good (Fig 8F). This is exhibited in the permanent collection of the National Archaeological Museum.

Although both graves were considered Chalcolithic by the Leisners [40], according to the documentation held at the National Archaeological Museum, Siret considered they might have been reused in later periods–an issue studied in depth by Lorrio [43]. In both cases, we cannot confirm whether the amber pieces correspond to the Chalcolithic dates or subsequent reuse.

#### Llano de la Teja 18 (Fonelas, Granada)

This is a mound with a quadrangular chamber with dimensions 1.8 x 1.7 m and a 2 m long corridor in which the remains of an individual and an amber bead are documented [40]. These are now at the National Archaeological Museum. The amber piece is ovoid, approximately 2.2 cm in length and 1.8 cm in maximum width, and is very degraded, although a core of vitreous appearance can still just about be observed.

Although the Leisners place this tomb within the Chalcolithic period, Siret places it within the Iron Age because of the metallic materials found '0.5 m from the floor', which leads us to think that a subsequent reuse process took place, as might be further evidenced by the presence of bronze bracelets (8–9% Sn) and silver beads [43]. The amber bead is depicted next to the corpse, two bracelets and a bronze earring in a drawing of the tomb's floor made by P. Flores (see Fig 120 in [43]), which suggests that the bead was deposited, along with the metallic grave goods, at the time of reuse. However, the precise context of the amber bead is not detailed in the description of the tomb, so we cannot know for sure whether the amber bead corresponds to a Chalcolithic dates or subsequent reuse.

#### Valle de las Higueras (Huecas, Toledo)

The site of Valle de las Higueras is the largest necropolis of hypogea found so far in the interior part of the Iberian Peninsula. With a C14 series between 2831–1511 cal. BC, eight funerary caves with collective burials have been documented [44]. In these caves, four amber beads from caves 1 and 3 were recovered [44, 45], in addition to the largest sets of perforated variscite and *Trivia arctica* in the whole of the interior Iberian Peninsula.

- Cave 1 has a chamber with a diameter of more than 3 m, with a small passageway on the side that gives access to another smaller chamber. To the West of the main chamber, a niche was dug in the ground where variscite necklace beads and ceramic remains were located. Also in the main chamber variscite and amber beads were recovered, along with Bell Beaker ceramic and two points of flint with cinnabar remains. The radiocarbon date from bones yielded a date between 2470–2030 cal. BC [45]. In the main chamber three amber beads were found in a very poor state of preservation. Only one allowed us to establish that it was a cylindrical bead 0.5 cm long, currently fragmented into two parts (Fig 8G). This bead was first analysed by S. Domínguez-Bella [46], whereas we have analysed the powdery remains of another specimen.
- Cave 3 is the most central one in the necropolis. It has a circular chamber with a narrow corridor that connects it to a rectangular antechamber with rounded sides. At the back of the chamber three niches were documented—they had been dug into the rock and had the most significant grave goods. In the West niche, two children were buried, in the Central niche an adult woman with two children in her arms, and in the East niche, a young adult and another older adult. In front of the niches a dozen individuals were buried, of which only one woman had grave goods, which comprised of a variscite necklace. At least twelve burial sites were documented in the antechamber. A cylindrical amber bead 1.3 cm in length and with a 0.2 cm perforation comes from this set (Fig 8H) and was analysed by S. Domínguez-Bella [46]. The dates obtained from these individuals place them in the second half of the 3^rd^ Millennium BC, between 2460–2190 and 2180–2140 cal. BC [45].

On the basis of his analysis, Domínguez-Bella ruled out a Baltic source but did not go on to propose an alternative origin [46]. In this work, we present the analysis of the remains of another of the beads from Cave 1, which has allowed us to propose a precise origin.

#### The artificial cave of Sao Paulo (Almada, Portugal)

The artificial cave of Sao Paulo is located in the Portuguese municipality of Almada, on the left bank of the Tagus River. This is a monument dug into the rock, and is part of a necropolis of which only two graves are known to be in artificial caves, although it is possible that the pressure of urban development has destroyed other similar graves. The artificial cave was discovered in 1988 and was excavated by the Department of Archaeology and History of the Municipal Museum of Almada. The hypogeum was dug in calcareous rock and consists of an elliptical chamber, almost circular, 7–7.5 m in diameter preceded by a corridor about 2.5 m long [47]. Inside the artificial cave, at least 254 individuals were documented [48] with a large amount of votive and adornment objects such as baetyls, idol plaques, green beads, gold sheets, etc. (e.g. [49]) that can be mostly ascribed to the 3^rd^ Millennium BC, although at higher levels there is documented evidence of reuse in the Iron Age and even Roman times. Dating carried out twice on human bone [49], directly provides dates of 2928–1921 and 2561–2141 cal. BC.

In the course of the excavation, two amber beads were identified in the lower strata, corresponding to the 3^rd^ Millennium BC. They can be found at the Municipal Museum of Almada.

The first bead is cylindrical and flattened, about 1.5 cm in diameter with a fine central perforation (Fig 8I). The second bead, about 1.8 cm in length, is fragmented, although we can intuitively discern an ovoid shape with a central longitudinal perforation (Fig 8J). In both cases, and especially the second one, the beads have significant surface degradation, are covered by a thick opaque layer, and are very crackled and altered by oxidation. However, both beads retain a core with the vitreous and translucent appearance typical of amber. In both cases, samples were taken of both the layer of oxidation and the more vitreous core.

#### Quinta do Marcelo (Almada, Portugal)

The Quinta do Marcelo archaeological site is located next to the mouth of the Tagus and has been interpreted as one of the first settlements established for exchanges with the Phoenicians [50]. However, the presence of iron in strata considered pre-Phoenician, with materials characteristic of the Late Bronze Age and dated between the 12^th^ and 8^th^ centuries BC [51], raises the prospect of a possible settlement before the arrival of the Phoenicians, and precolonial contacts and exchange [51, 52]. The site has also been considered a seasonal camp dedicated to gold mining in the sands of the Tagus because of the potential presence of a cupel, possible lithic mining tools and gold and mercury residues documented at the bottom of a receptacle [53, 54].

The materials are held at the Municipal Museum of Almada, largely unpublished, with partial references in more general works. The amber bead that we are concerned with appeared in a waste pit or "bag 2", together with very high-quality materials characteristic of the Late Bronze Age: fine ceramics with internal and external decoration [50, 54], two fibulae and three pieces of iron [51].

The amber object is a circular bead of approximately 1.1 cm in diameter, with a central perforation. Although it has light oxidation on the surface, its state of conservation is considerably good (Fig 8K).

## Results

The FTIR spectra obtained for the different samples allow us to confirm that, in all cases, the raw material used is amber. At the same time, they present significantly different patterns, which allow us to note the use of amber with different origins, and exposed to different postdepositional conditions. At least three different geological sources can be identified, which are discussed in turn:

### Iberian origin

**Fig 10:**
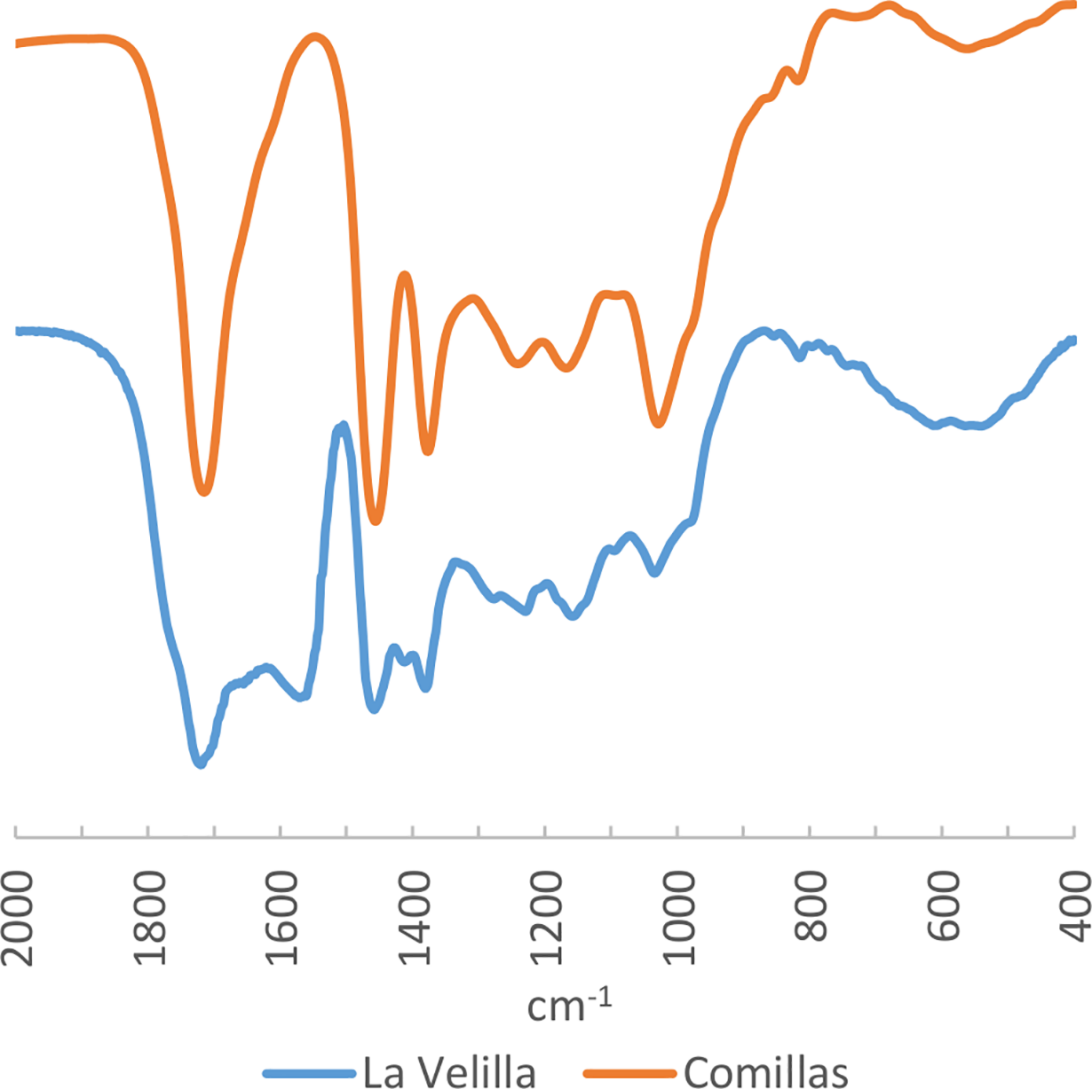
FTIR Spectra of amber sample from La Velilla compared to reference Cretaceous Iberian amber from Comillas.

The only object for which we can propose a peninsular source is the bead from La Velilla (Palencia), the archaeological site with amber that is closest to some of the Iberia’s geological amber deposits. In this case, we can see that both the spectrum of the bead and the characteristic spectra of Cretaceous Iberian amber display similar patterns in the diagnostic area of the spectrum (i.e. c. 1600–900 cm^-1^). The amber spectrum from La Velilla is characterised by five well-defined bands: at 1570 ±5 cm^-1^ by the aromatic double bonds C = C, at 1457 ±5 and 1381 ±5 cm^-1^ attributable to alkyl groups, the first the -CH^2^ and -CH^3^ flexures (bending, δ) and the second due only to the -CH^3^ flexure; a band at 1158 ±5 cm^-1^ which can be attributed to the tension of the C-O simple ester bond, and one last band at 1034 ±5 cm^-1^ as well as three secondary peaks at 1411 ±5, 1277 ±5 and 1230 ±5 cm^-1^ (e.g. [55]) (Fig 10; Table 3).

**Table 3:** Main bands cm^-1^ from amber samples analysed with a possible Iberian provenance.

Sample	1570 ±5 cm^-1^	1500–1300 ±5 cm^-1^	1300–1100±5 cm^-1^	1030±5 cm^-1^	Reference
La Velilla	1570	1457 1411(sec.) 1381	1277(sec) 1230 (sec) 1158	1034	[This paper]
Morín Cave^a^	1570	1457 1410(sec) 1380	1230 1159	1030	[8]
El Pendo	1570	1457 1410(sec) 1380	1230 1159	1030	[8]
La Garma	1570	1457 1410(sec) 1380	1230 1159	1030	[8]
Trikuaizti I		1457 1380	1229 1159	1030	[8]
Los Lagos I		1458 1379	1228 1157	1031	[56]
*Reference Cretaceous Iberian amber*		*1457* *1376*	*1226 (sec)* *1155*	*1030*	*[19; this paper]*

Sec. = Secondary; s.s. = soft shoulder. Peaks deemed to be altered by oxidation processes are highlighted in grey.

^a^Not all the peaks are specifically reported for samples from Morín Cave, El Pendo and La Garma in Álvarez et al. [8].

This spectrum is similar to the reference spectrum of the Cretaceous Peninsular amber described above, which is characterised by bands at 1457, 1376, 1155, and 1030 ±5 cm^-1^ as well as a secondary peak at 1226 ±5 cm^-1^. The most significant differences in the spectra are the band at 1570 ±5 cm^-1^ and the secondary peak at 1411 ±5 cm^-1^ in the sample from La Velilla, which do not appear in the geological samples, and may be due to oxidation processes (as seen in other samples taken from the weathered layers of amber; see below). In fact, the spectrum from La Velilla sample is almost identical to the archaeological amber samples from the Palaeolithic settings of La Garma A [57], for which a local origin, the Cretaceous amber of the North of the Iberian Peninsula, was proposed. We can also therefore propose that this archaeological amber has an Iberian origin.

#### Baltic origin

**Fig 11:**
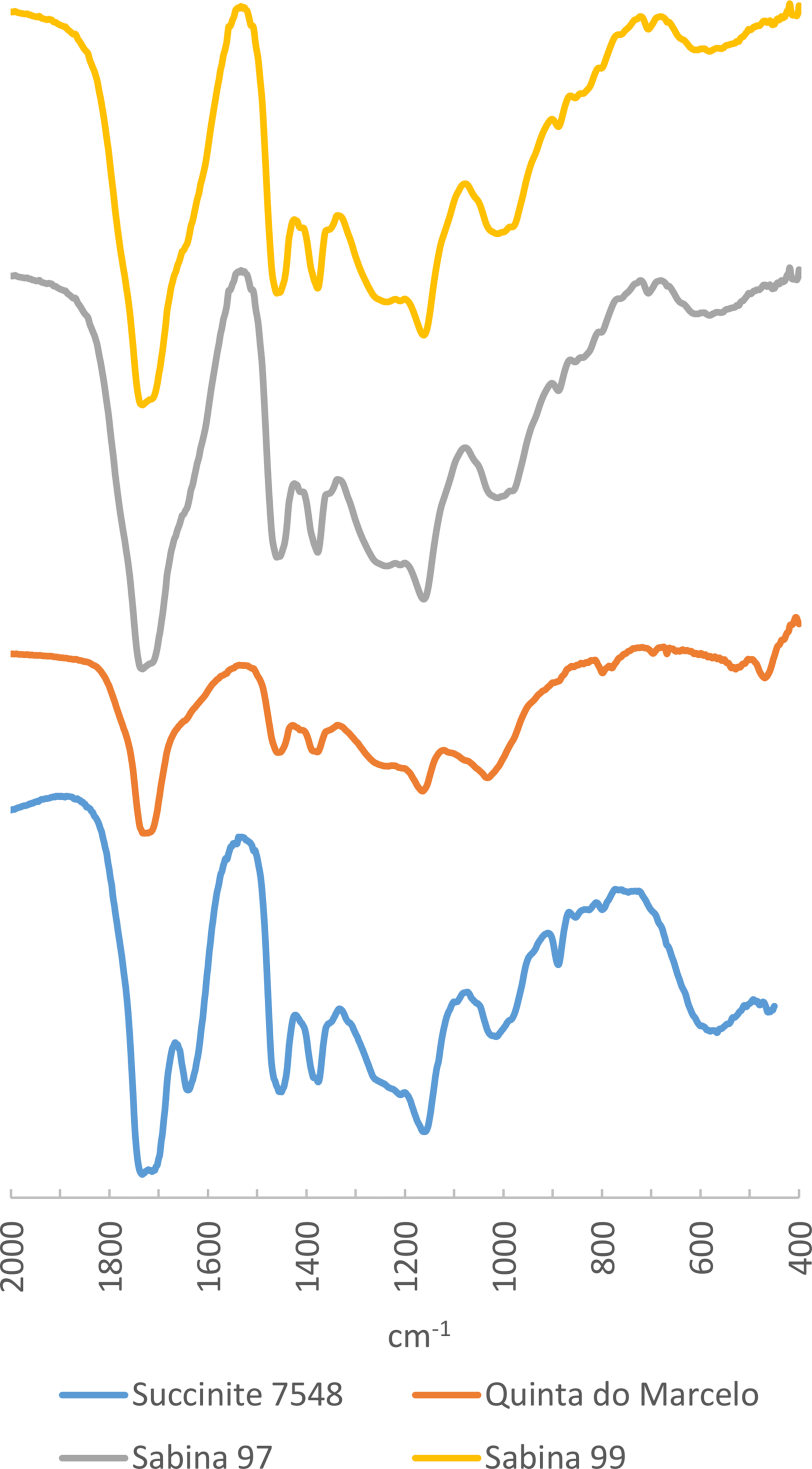
FTIR Spectra of amber samples from Quinta do Marcelo and Llano de la Sabina 97 and 99 compared to reference spectrum of Baltic succinite.

The characteristic spectrum of Baltic amber is very well known [58–60], which has allowed the recognition of the Balstic sources of as the most heavily exploited since the Bronze Age at least. Its FTIR spectrum is characterised by a strong absorption peak at 1160–1150 cm^-1^ preceded by a horizontal band between 1250 and 1180 cm^-1^ –the so-called ‘Baltic shoulder’. This feature has been documented in only three of the samples studied for this paper: the beads from Llano de la Sabina 97 and 99, and Quinta do Marcelo. In all three cases, the Baltic shoulder can be clearly identified, so we can be confident about their attribution to an exogenous origin (Fig 11; Table 4).

**Table 4:** Main bands cm^-1^ from amber samples analysed with a possible Baltic provenance.

Sample	1500–1300 ±5 cm^-1^	1250–1180 ±5 cm^-1^ *(Baltic shoulder)*	1160 ±10 cm^-1^	Reference
Llano de la Sabina 97	1456 1377	1247–1180	1162	[This paper]
Llano de la Sabina 99	1457 1381	1242–1180	1163	[This paper]
Quinta do Marcelo	1455 1381	1245–1180	1165	[This paper]
*Reference Baltic succinite*	*1451* *1376*	*1250–1180*	*1160*	*[58–60*, *this paper]*

#### Sicilian origin

**Fig 12:**
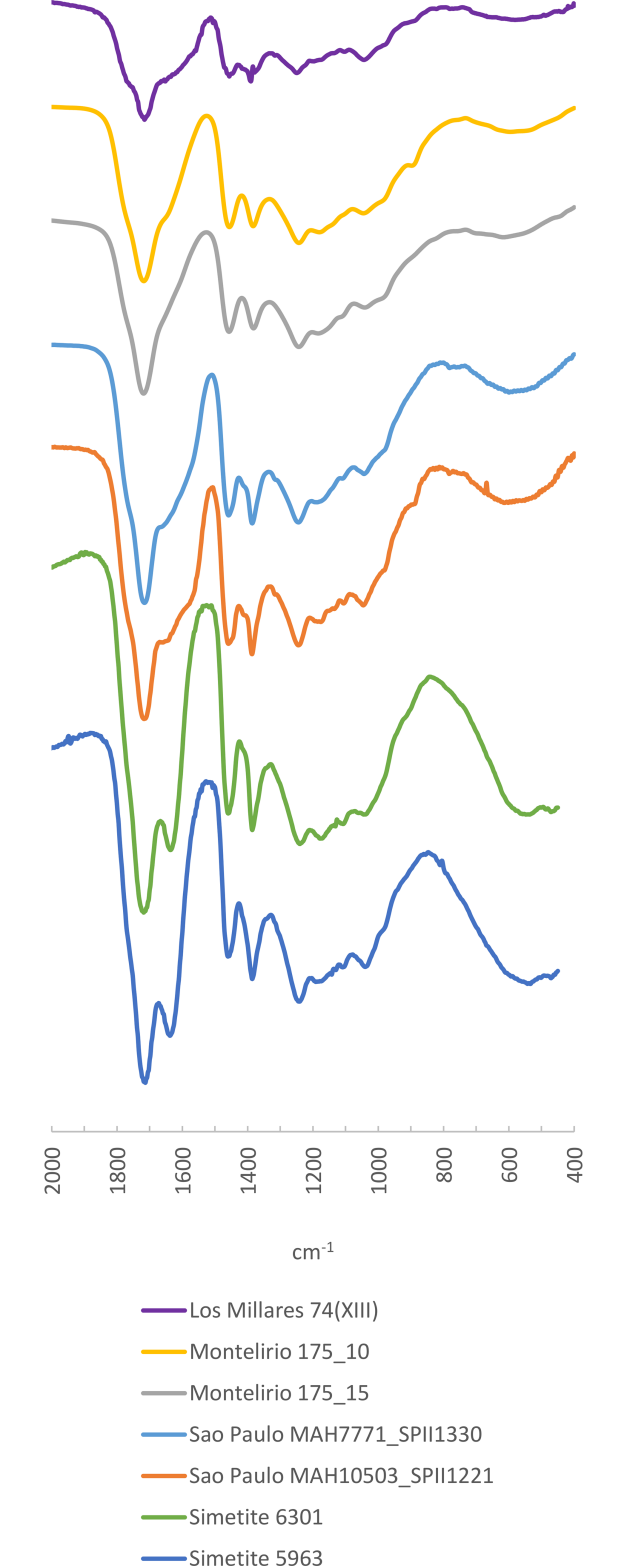
FTIR Spectra of amber samples from the artificial cave of Sao Paulo, Mound 74(XIII) from Los Millares and the tholos of Montelirio compared to reference spectra of Sicilian simetite.

A Sicilian origin has already been proposed for some artefacts from the site of Valencina de la Concepción [9, 12]. One of us recently conducted a study of more than 250 pieces from the tholos of Montelirio, sampling separately the weathered surface and the vitreous core, and matching the spectrum of the amber core with the spectrum that is characteristic of Sicilian simetite [12]. We repeated the same procedure for the beads from the artificial cave of Sao Paulo and the Mound 74(XIII) from Los Millares and obtained similar results: the amber core spectrum of these samples, as in the case of the tholos of Montelirio, is characterised by bands at 1456 and 1382 ±5 cm^-1^, normal in old resins, which correspond to the -CH^2^ and -CH^3^ flexures [55]; a third band at 1242 ±5 cm^-1^ followed by a secondary peak of lower intensity at 1180 ±5 cm^-1^ and a second peak, also of low intensity, at 1040 ±5 cm^-1^ that would correspond to the vibrations of the simple C-O and C-C bonds [55]. In one sample from the artificial cave of Sao Paulo and some from the tholos of Montelirio a slight shoulder is observed at 890 ±5 cm^-1^ which could be a consequence of the degradation of the components of the exocyclic methylene group in the resin. Guiliano et al. [61] have shown that this peak disappears if the amber undergoes thermal exposure, so its presence or absence cannot be considered in terms of provenance, but rather in terms of conservation, diagenesis or the postdepositional environment to which the amber had been exposed (Fig 12).

**Fig 13:**
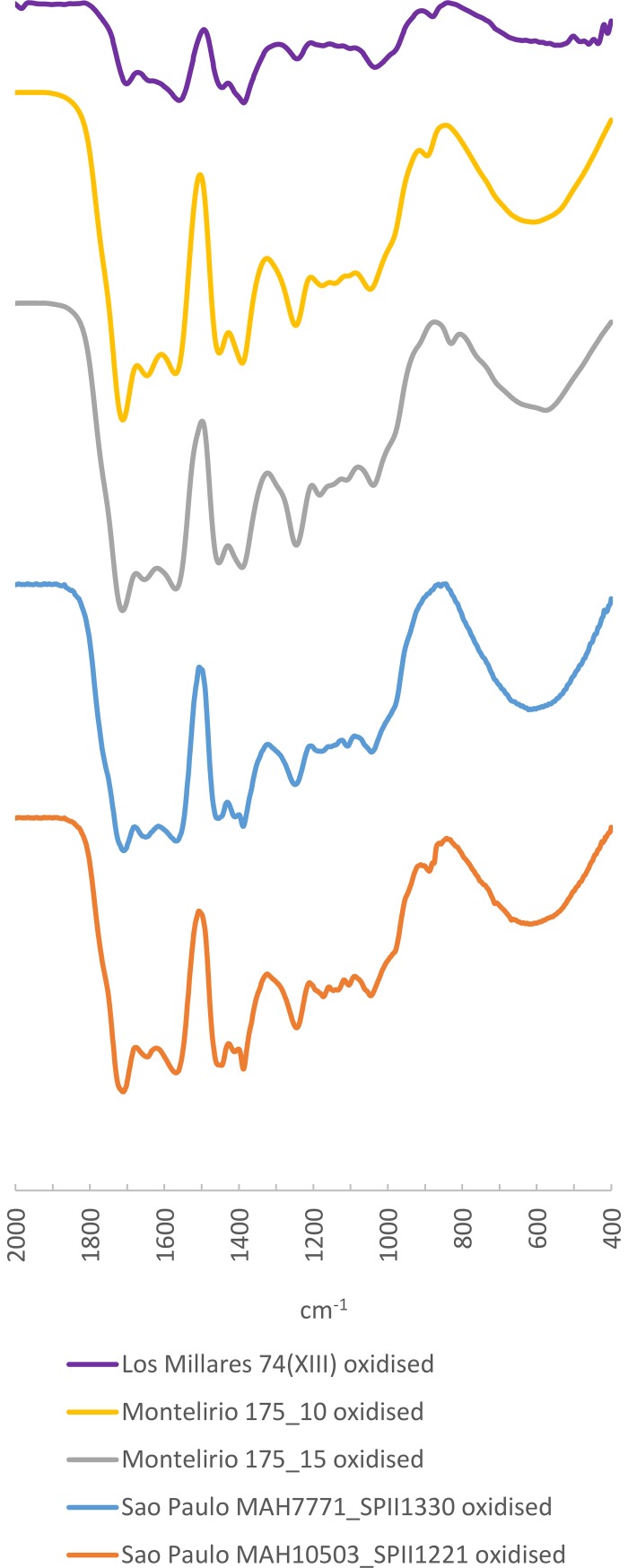
FTIR Spectra of the weathered layers of the same amber samples from the artificial cave of Sao Paulo, Mound 74(XIII) from Los Millares and the tholos of Montelirio.

The spectra of the weathered surface of these same samples present significantly different features in the area between 1800 and 900 cm^-1^ (Fig 13). In these cases, as with the samples from the tholos of Montelirio, we observed a secondary absorption doublet at 1646 ±5 and 1570 ±5 cm^-1^ whereas in the core amber samples only a slight shoulder is observed. These bands, which correspond to the formation of salts and C-O stretching, have also been documented in the analyses of oxidation layers of Baltic amber (e.g. [62]), although the diagnostic ‘Baltic shoulder’ is still distinguishable in these. The subsequent bands remain the same both in the core amber and in the weathered layer, although the band at 1456 ±5 cm^-1^ has a significantly lower intensity in the weathered layer; after the secondary peak at 1180 ±5 cm^-1^, which also has a lower intensity, there is a second peak of very low intensity, which in some samples only appears as a slight shoulder at 1143 ±5 cm^-1^. The shoulder observed at 890 ±5 cm^-1^ is absent from the spectra of weathered samples both from the tholos of Montelirio, Mound 74(XIII) from Los Millares and the artificial cave of Sao Paulo. Guiliano et al. [61] proposed that this peak disappears with thermal exposure; hence it seems it cannot be used for provenance and its absence may be due to postdepositional alteration.

**Fig 14:**
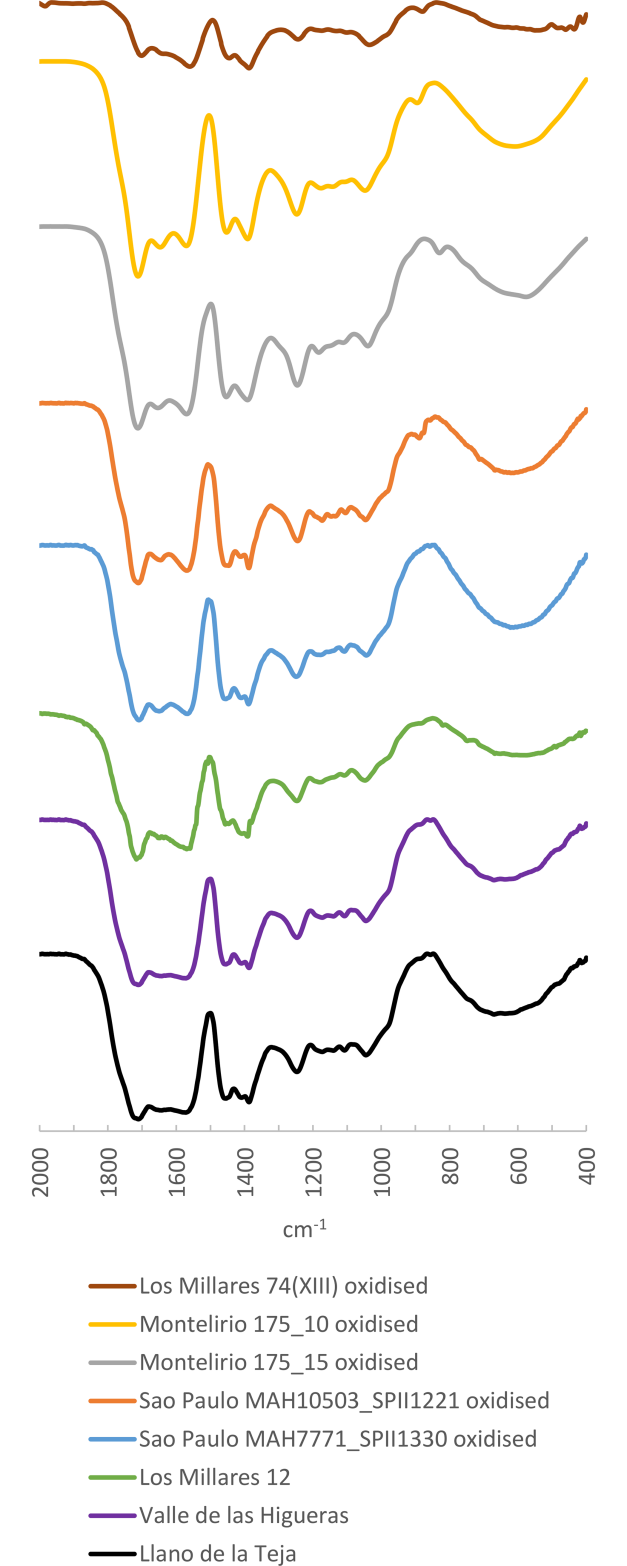
FTIR Spectra of amber samples from Llano de la Teja 18, Valle de las Higueras and Mound 12 from Los Millares compared to weathered layers from the artificial cave of Sao Paulo, Mound 74(XIII) from Los Millares and the tholos of Montelirio.

As a matter of fact, this same pattern is also observed in some of the most degraded samples elsewhere, such as those from Valle de las Higueras, Llano de la Teja 18 and Mound 12 from Los Millares (Fig 14; Table 5), which yielded spectra very similar to those from the weathered layers of the beads from the tholos of Montelirio, the Mound 74(XIII) from Los Millares and the artificial cave of Sao Paulo, and to that of an amber bead from Los Cuarenta Cave, a sepulchral cavity in Córdoba dating to the second half of the 4^th^ Millennium BC [63].

**Table 5:** Main bands cm^-1^ from amber samples analysed with a possible Sicilian provenance.

Sample	1640±10 cm^-1^	1570 ±10 cm^-1^	1450–1390 ±5 cm^-1^	1245±5 cm^-1^	1175 ±5 cm^-1^	1105 ±5 cm^-1^	1045±5 cm^-1^	890 ±10 cm^-1^	Reference
Valle de las Higueras	1638	1562	1454 and 1413(sec.) 1385 (s.s.)	1249	1177 (sec.)	1105 (sec.)	1040	880 (sec.)	[This paper]
Llano de la Teja 18	1640 (s.s.)	1575	1455, 1409 and 1388 (sec.)	1247	1175 (sec.)	1107 (sec.)	1043		[This paper]
Los Millares 12	1649 (sec.)	1568	1449 (sec.), 1409(s.s.) 1394	1247	1179 (sec.)	1108 (sec.)	1048	880 (s.s.)	[This paper]
Los Millares 74(XIII) (weathered layer)	1643 (sec)	1561	1445 (sec), 1386	1243	1174 (sec)	1101 (sec)	1034	881	[This paper]
Sao Paulo MAH 10503 (weathered layer)	1648 (sec.)	1570	1451 and 1389 (sec.)	1245	1175 (sec.)	1108 (sec.)	1047	892 (sec.)	[This paper]
Sao Paulo MAH 7771 (weathered layer)	1651 (sec.)	1570	1453 and 1390 (sec.)	1249	1181 (sec.)	1110 (sec.)	1044		[This paper]
Los Cuarenta Cave	1648 (sec.)	1570	1451 and 1392 (sec.)	1248	1177 (sec.)	1107 (sec.)	1046		[63]
Tholos of Montelirio (weathered layer)	1653 (sec.)	1569	1453 and 1391 (sec.)	1245	1182 (sec.)	1111 (sec.)	1040	894 (sec.)	[12]
Tholos of Montelirio (amber)	1640 (shoulder)		1456 and 1383	1242	1180 (sec.)		1045		[12]
Los Millares 74(XIII) (amber)	1640 (shoulder)		1450 and 1382	1237	1173	1105 (sec)	1036	889 (sec)	[This paper]
Sao Paulo MAH 10503 (amber)	1640 (shoulder)		1454 and 1385	1244	1183 (sec.)		1047		[This paper]
Sao Paulo MAH 7771 (amber)	1640 (shoulder)		1456 and 1384	1244	1180 (s.s.)		1044		[This paper]
Dolmen of La Pastora	1640 (shoulder)		1458 and 1392 (1374 sec.)	1238	1172		1041		[This paper]
Los Millares 63(III)	1640 (shoulder)		1455 and 1398 (1376 s.s.)	1249	1175 (s.s.)		1043		[This paper]
Chousa Nova	1635		1457 and 1386	1243	1174	1106	1019		[64]
Dolmen de Alberite	1650		1450 and 1375	1242	1195	1145	1090		[65]^a^
*Reference Sicilian simetite*	*1637 (sec*.*)*		*1457 and 1386*	*1239*	*1178 (sec*.*)*		*1041*		[66 This paper]

Sec. = Secondary; s.s. = soft shoulder. Peaks deemed to be altered by oxidation processes are highlighted in grey.

All in all, we therefore assume that in all of these cases the original amber would also give spectra similar to those observed in the samples of the vitreous amber cores from the tholos of Montelirio, Mound 74(XIII) from Los Millares and the artificial cave of Sao Paulo. These spectra differ significantly from those of Cretaceous Iberian amber, and they lack the characteristic Baltic shoulder observed–while they retain the typical bands of Sicilian simetite (Fig 12). At present, simetite remains the only known amber spectrum that resembles these archaeological samples, and hence we assign them to a Sicilian origin.

**Fig 15:**
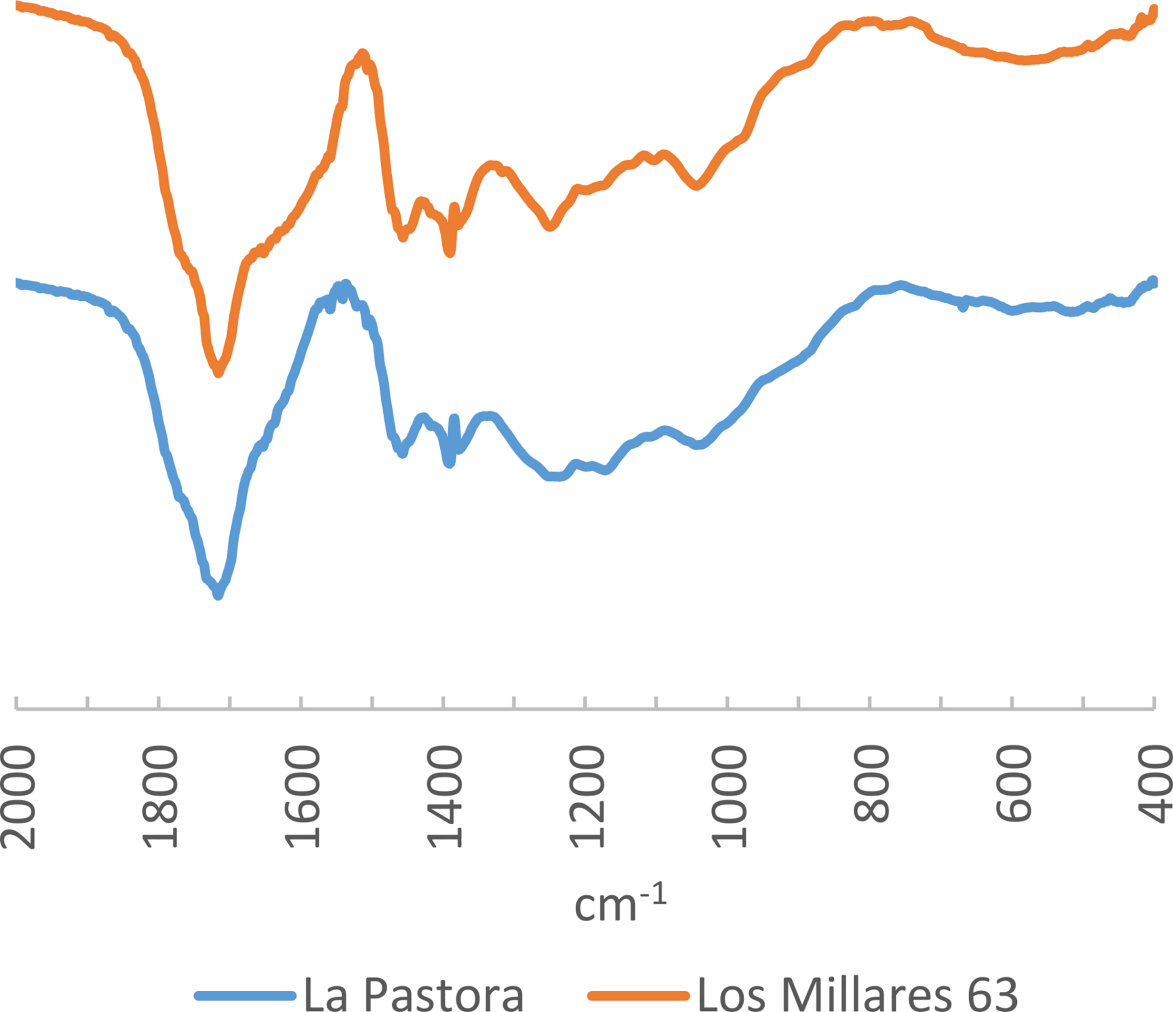
FTIR spectra of samples from Dolmen of la Pastora (Valencina de la Concepción) and Mound 63(III) from Los Millares.

Lastly, the analysed samples from the Dolmen of La Pastora in Valencina de la Concepción and the beads from burial Mound 63(III) from Los Millares produced very similar spectra, suggesting that they were made of amber of the same origin (Fig 15). None of them has bands at 1640 and 1570 ±5 cm^-1^ and they are both characterised by peaks at 1455 and 1392 ±5 cm^-1^ followed by a secondary peak at 1375 ±5 cm^-1^, as well as bands at 1240 and 1043 ±5 cm^-1^. Notably, the characteristic bands of Cretaceous Iberian amber are not present.

**Fig 16:**
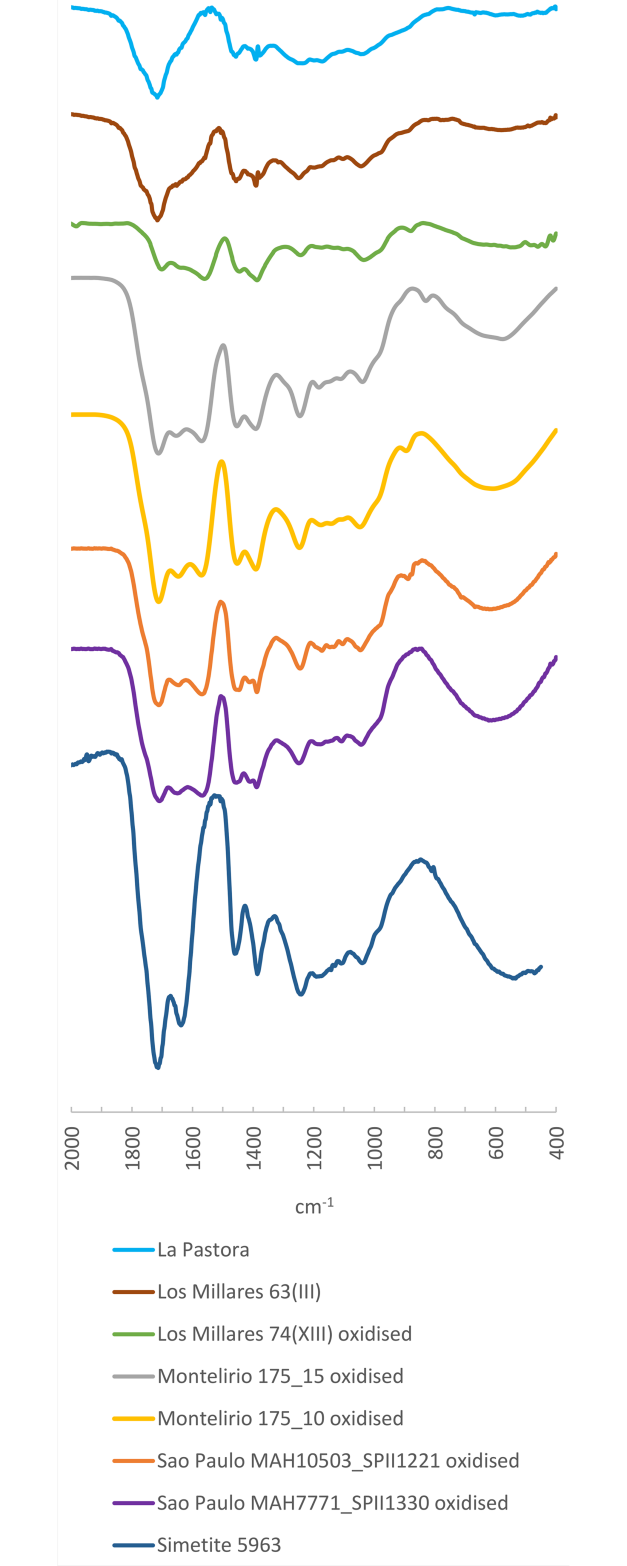
FTIR Spectra of samples from Dolmen of la Pastora (Valencina de la Concepción) and Mound 63(III) from Los Millares compared to weathered layers from the artificial cave of Sao Paulo, Mound 74(XIII) from Los Millares and the tholos of Montelirio as well as the simetite reference spectrum.

The spectra for both samples show some similarity with that of the simetite reference (Fig 16), although the presence of a slight secondary absorption peak at 1376 cm^-1^ obliges us to be cautious when proposing Sicily as the source: this band does not appear in other degraded samples, so we cannot confident dismiss it as a result of postdepositional alteration. However, at present, Sicily offers the most similar spectrum we know of. What we can be sure of is that it is exotic material, since it does not resemble the Cretaceous amber spectra of Iberia, where no workable amber deposits of other geological age have been discovered (amber occurs only disaggregated as tiny nodules of a few millimetres in the two Late Triassic amber localities shown in Fig 1, and hence it is unlikely to have constituted a suitable raw material for bead making).

## Discussion

The new results presented in this article provide a stronger basis to confirm and expand the diachronic perspective of changes in the provision and exchange of amber in the Iberian Peninsula from the Late Palaeolithic period to the Late Bronze Age [9].

**Fig 17:**
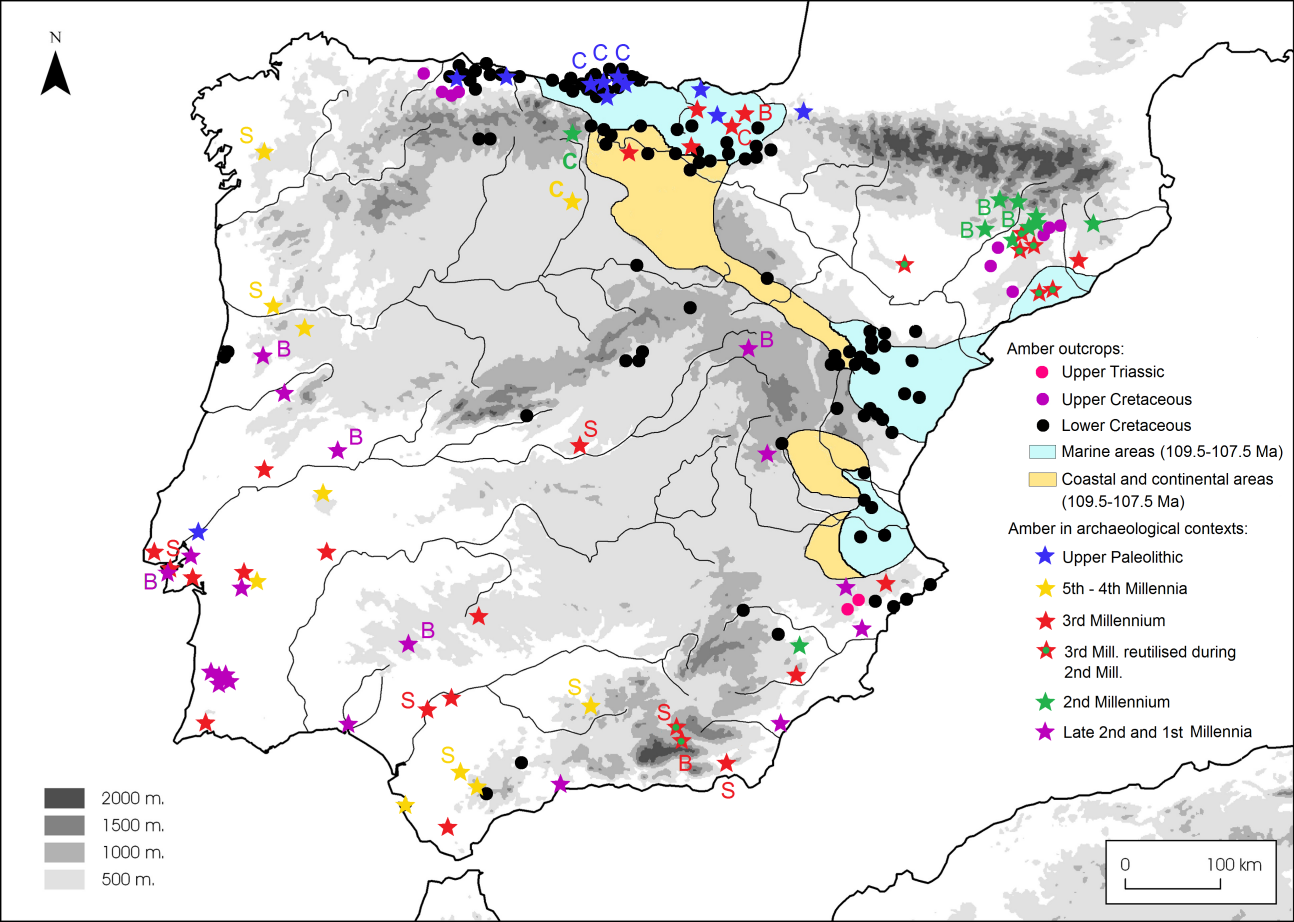
Distribution of amber objects in Iberia and their proposed geological origins. C = Cretaceous (Iberia); S = Simetite (Sicily); B = Succinite (Baltic).

For the Late Palaeolithic, amber consumption is already well documented, although its distribution is constrained to the Northern part of Iberia and the origin of the raw material is local in all cases (Fig 17). In the rest of the Peninsula, amber objects only begin to appear in the 5^th^–4^th^ Millennia BC, especially in the South and West, concurrently with the arrival of exogenous amber. We now have analytical support for this phenomenon in the samples from Chousa Nova, Mamoa V of Chã de Arcas, Dolmen of Alberite, Campo de Hockey, Los Cuarenta Cave and La Velilla [16, 63, 64, 67, 68, this paper].

Of these samples, the beads from the Dolmen of Alberite (6^th^–5^th^ Millennia BC) were the first to be explicitly identified as simetite in several publications [16], and hence purported to be Sicilian in origin. Recently, in an interesting article about the origin of amber recovered in Italy, Angelini and Bellintani have questioned the Sicilian origin of the Alberite samples by noting that the original publication shows a “totally different spectrum in the fingerprint region from the typical spectrum of simetite” [69]. While the shape of the FTIR spectrum shown in a relatively recent publication [16] does superficially resemble that of reference simetite, and it is here that the authors first propose this identification (see also [67]), the location of the absorption bands reported in an earlier publication [65] does not accurately match those in our reference simetite sample (see Table 5), and hence a Sicilian origin is possible but not conclusively proved.

In any case, FTIR spectra matching those of simetite have also been published in the studies of the Mamoa V of Chã de Arcas and Chousa Nova (5^th^ Millennium BC) [68]. The latter study has also been questioned by Angelini and Bellintani [69] because of the presence of an absorption peak at around 1000 cm^-1^. Although it is true that the peak to which they refer appears displaced (at 1019 cm^-1^ instead of 1041 ±5cm^-1^ as it is in the reference spectra of simetite), all the bands described coincide with those of simetite (Table 5), and it therefore seems sensible to retain this assignation as the most plausible. Angelini and Bellintani [69] also cast doubts on the Sicilian origin of the amber pommel of the structure 10042–10049 of Valencina de la Concepción published in Murillo-Barroso and Martinón-Torres [9], because of “the presence of two absorption peaks around 1000 and 888 cm-^1^”, although they recognise that a Sicilian origin is still plausible in this case. The first peak they refer to is actually at 1045 (simetite = 1041 ±5 cm^-1^). The second peak (887cm-^1^) cannot be considered a marker of provenance but of postdepositional alteration since, as noted above, this band disappears following thermal exposure [61].

Other deposits of Late Neolithic chronology and bearing amber include the Campo de Hockey analyses (transition 5^th^–4^th^ Millennia BC); the results of these analyses have not been published, although it has been confirmed that the amber is not Baltic succinite [67]. In addition, we have the samples from Los Cuarenta Cave [63] and La Velilla. As noted above, the (degraded) samples from Los Cuarenta Cave yielded identical spectra to the weathered layers analysed in other amber objects from the 4^th^–3^rd^ Millennia BC, such as those from the tholos of Montelirio or the artificial cave of Sao Paulo, whose unaltered cores also give the same spectrum as Sicilian simetite. We therefore propose that those samples constitute degraded simetite [63].

Regarding the interior part of the Peninsula, the earliest data concerning the presence of amber in the Northern Plateau (La Velilla) points to an Iberian source and thus confirms the strength of regional networks. The use of regional raw materials in the North Peninsular area continued until the 2^nd^ Millennium BC, as demonstrated by the amber beads from Los Lagos I [8].

The use of these local resources, however, is not documented in the Southern part of the Iberian Peninsula. Outside the Northern region, where geological amber deposits are abundant, the amber objects analysed are, in all cases, foreign. This preference for exogenous raw materials could respond to different causes that are not mutually exclusive. From a technological point of view, the greater age of Iberian amber makes it more fragile and therefore more difficult to carve than succinite or Cenozoic simetite. From a more social perspective, it is worth considering that amber objects may have arrived in Iberia already manufactured; in this case, the choice of foreign objects would be driven not so much (or not just) by the quality of the material, but by the role played by these amber objects in establishing social relations or alliances through exchange or gift policies.

This presence of foreign amber materialises in a much more evident way during the 3^rd^ Millennium BC [9, 70]. This increase in foreign amber is noticeable not so much in the size of the assemblages (the 250 beads from the tholos of Montelirio in Valencina de la Concepción being an exception to the general trend of only a few beads per deposit), as in the number of sites containing amber, which practically doubles compared to the previous period. The presence of Sicilian simetite is confirmed in 3^rd^ Millennium BC contexts for the objects from Valencina de la Concepción and those from the artificial cave of Sao Paulo, Valle de las Higueras, Llano de la Teja 18 and Los Millares.

In the South Plateau, the necropolis of Valle de las Higueras is an exception because of the presence of amber associated to Bell Beaker, something not found in the area’s hypogea [71], nor in those of the North Plateau. Valle de las Higueras points to a mixed situation where the variscite comes from an Iberian source in Zamora, the *Trivia arctica* is from the Atlantic and the amber from Sicily, an image that is fairly coherent with the central location of this necropolis in the Iberian Peninsula.

Overall, while the sites in the North of the Iberian Peninsula show continuity and the survival of the local networks of exchange, the identification of Sicilian amber in Andalusia demonstrates the beginning of Mediterranean exchange circuits dating back to the 4^th^ Millennium BC at least; in parallel, the presence of variscite from the Iberian West in the megaliths of Luffang (North-West France) dated to the 4^th^ Millennium BC [7] confirms the existence of Atlantic exchange routes. The role of the South in the intensification of social and economic complexity of the 3^rd^ Millennium BC is notable and reflected in the greater participation of this region in trans-regional circuits of exchange, as manifest in the fact that all the sites with more abundant amber and other ‘exotic’ objects are located in this area.

Thus we can confirm the presence of simetite in the Iberian Peninsula as early as the 4^th^ Millennium BC, and possibly earlier. Interestingly, the first amber objects recovered in Sicily and assigned a local origin also date from the 4^th^ Millennium BC [69, 72]. However, there is no other evidence that might indicate direct contact between Sicily and Iberia. Instead, we do know about the links between the Iberian Peninsula and North Africa, and the fact that present-day Tunisia was involved in Central Mediterranean exchange networks of obsidian. The island of Pantelleria, situated between the shores of Tunisia and Sicily, appears to have been the main source of obsidian for North Africa, Tunisia, Sicily and Italy [2, 3]. Even though no obsidian artefacts are known in Southern Iberia, it seems plausible that Sicilian amber reached the Iberian Peninsula indirectly through exchanges with North Africa. In fact, the spatial distribution of amber findings from the 4^th^ and 3^rd^ Millennia BC is similar to that of ivory objects [73], which could suggest that both materials reached the Iberian Peninsula following the same or similar channels.

The steady increase in amber consumption we had been observing since the 5^th^ Millennium BC seems to be interrupted abruptly in the 2^nd^ Millennium BC. With the exception of very late examples addressed below, there are only a few contexts from the 2^nd^Millennium BC in which amber is documented, and these are more or less restricted to the North-Eastern part of Iberia. It is in this period, however, when we first document Baltic succinite, which from then on will become virtually the only raw material used. A similar situation, namely simetite being the only type of amber deposited, has been described for Italy and Sicily from the Eneolitic to the Middle Bronze Age [69]. It should be noted, however, that Baltic amber is first documented in Sicily slightly earlier (1600–1300 cal. BC) than in Iberia; furthermore, in contrast with Iberia, the frequency of amber is prominent in the Eastern and Central Mediterranean during the 2^nd^ Millennium BC (especially in the Mycenaean Culture and the Peloponnese), although most of the objects, especially in Early Mycenaean contexts, are concentrated in a few of the richest graves [74–76]. Sicilian simetite continues to be used in Eastern and Central Mediterranean, even if with less intensity, as evidenced by the 2^nd^ Millennium BC samples from Greece analysed by Beck and Hartnett [66] and the sample from Madonna del Piano (Catania, Sicily) dated to the 10^th^ century BC [69]. However, most of the amber documented in Greece and Italy during the second half of the 2^nd^ Millennium BC is Baltic in origin, evidencing connections between Nordic/Atlantic Europe and the Mediterranean (e.g. [75–77]).

Unfortunately, the lack of absolute dates in the Bronze Age settings of the North-East part of the Iberian Peninsula where amber is documented prevents us from accurately pinpointing the moment when succinite begins to reach Iberia. The earliest known evidence of Baltic amber may be that of the Dolmen of Larrarte (Guipúzcoa). Published dates for this site range between 5345–4051 cal. BC and 4236–3542 cal. BC [78]. However, these dates were obtained from charcoal samples collected outside the chamber, as human remains did not have sufficient collagen for dating. The specific context where amber was recovered remains undated although the grave goods recovered are characteristic of the Copper Age: Bell Beaker pottery, lithics and several beads including green and amber beads. In this particular case, the supra-regional Bell Beaker network may have facilitated the acquisition of amber; however, we should emphasise that on the basis of the current evidence we cannot confirm a direct association between Bell Beaker phenomenon and Baltic amber in Iberia: out of 35 archaeological sites of this chronology with amber objects, only 11 yielded Bell Beaker pottery, and up to now, FTIR analyses have only been conducted on 3 of these sites: Larrarte (Baltic), Trikuaizti I (local) and Valle de las Higueras (Sicilian). A systematic analysis of the remaining sites is necessary to investigate the possible significance of this network for the early arrival of Baltic amber to Iberia.

In the Iberian North-East, Baltic amber has been documented in Pedra Cabana, Cabana del Moro de Colomera and Muricecs [9, 24]. Although we lack absolute dates from these contexts, Rovira i Port [24] considers that Pedra Cabana and Cabana del Moro de Colomera should be dated to the “advanced Middle Bronze Age” (c. 1500–1300 BC) on the basis of artefacts types. The Muricecs deposit, also based on metal objects typology, has been tentatively dated to around 1400–1200 BC [79].

In the South-East, we have the samples from Llano de la Sabina 97 and 99, recovered in megalithic monuments with evidence of reuse, with mixed materials from both the 3^rd^ and the 2^nd^ Millennia BC. Archaeologically it is impossible to assign the pieces of amber to any particular chronology but their Baltic origin suggests that they correspond to the later reutilisations. The sample from Llano de la Teja 18, another monument with evidence of reuse, raises more doubts, as the FTIR spectrum for the amber is consistent with Sicilian simetite and hence would suggest a 3^rd^ Millennium BC chronology. We acknowledge, however, the risks of circularity in our argument.

The decline in amber consumption during the 2^nd^ Millennium BC is more significant when placed against the backdrop of other changes: during the 4^th^ and 3^rd^ Millennia BC, amber had been closely linked to megalithic monuments of undeniable symbolic significance, and often accompanied by other special raw materials such as ostrich eggs, ivory, cinnabar or gold; all of these exotic raw materials appear to vanish concurrently [80]. This radical change in the material repertoire may have responded to the need of new ideological and symbolic standards to legitimise the new social orders characteristic of Bronze Age societies. The symbolic expressions of the Chalcolithic period are left behind, and new codes are established where amber no longer plays an important role and is replaced, with increasing frequency, by metal objects. The examples we have observed of tholoi reuse, as in Llano de la Sabina 97 and 99, could well be framed as a response or resistance to the new ideological order and the survival of ancestral traditions (e.g. [81]). Similar transformations of materiality reflecting ideological changes have also been proposed for Mycenaean Greece where, contrary to Iberia, amber would have played a major role: firstly in the construction of Early Mycenaean warrior identity (c. 1700–1420 BC), to acquire later a cosmological significance during the Mycenaean post-palatial period (c. 1200–1060 BC) [74].

Although now with a Baltic origin (simetite is not documented again in subsequent chronologies), amber will reappear in Iberia in the Late Bronze Age and the Early Iron Age (Fig 17), when Mediterranean contacts again have an important impact on the societies of the Peninsula, as reflected at the sites of Quinta do Marcelo (12^th^-8^th^ centuries BC), Moreirinha (13^rd^-9^th^ centuries BC) and Senhora da Guia (10^th^-8^th^centuries BC) in Portugal [this paper, 68] or Palacio III (11^st^-6^th^ centuries BC) and Herrerías II (10^th^ century BC) in Spain [23, 82]. Dating the resurgence of the use of amber in Iberia, and particularly the onset of Baltic amber, is particularly important in the light of claims that Scandinavian trader-raiders engaged directly in exchange with Iberian communities to obtain metals, and that amber was the material supplied in return [83–85]. However, contextual associations are as important as dates: amber exchange cannot be studied in isolation, and Baltic provenance does not necessarily imply direct contact with the Nordic region, especially when we bear in mind that Baltic amber is also documented in Central and Eastern Mediterranean in this period and Mediterranean metals are documented in the Nordic region as well [77, 86]. It must be highlighted that the spatial distribution of amber objects from 12^th^ century BC is concentrated close to coastal sites, and the earliest evidence of Baltic succinite in Southern and Western Iberia occurs in association with some of the earliest iron objects, and is hence connected to a technology that likely arrived via the Mediterranean. This is the case of Quinta do Marcelo, Moreirinha, Senhora da Guía and Palacio III. Moreover, there are other objects which point to Mediterranean pre-colonial contacts: metal artefacts recovered at Quinta do Marcelo have been related to the Mediterranean [52]; bronze wheeled stands from Senhora da Guía are unequivocally linked to the Mediterranean, with Syrian-Cypriot or Sardinian style [87]; and silver objects from Palacio III do also correspond to Mediterranean traditions [82]. All of these materials must be contextualised in a period of Mediterranean contacts previous to the Phoenician colonisation. Some of the earliest examples of these contacts are the Mycenaean pottery recovered at Llanete de los Moros (Córdoba) dated to the 14^th^-13^th^ centuries BC or the wheeled and Cogotas I pottery from Cuesta del Negro (Granada) dated to the 14^th^ century BC, also with a proposed Mycenaean origin (see Torres Ortiz [88] for discussion of absolute dates of early pre-colonial contacts, and Celestino et al. [89] for general discussion on Mediterranean pre-colonial contacts).

In the North-Eastern Iberian region, continental contacts through the Pyrenees continued during the Bronze Age (as evidenced by the Muricecs deposit of Baltic amber) as well as Mediterranean ones, although the latter being seemingly less important. This panorama will change during the Late Bronze Age/Early Iron Age, when Mediterranean contacts become more intense [90]. Based on the current evidence, it would seem that Baltic amber only reaches Southern Iberia in any significant amounts towards the very end of the 2^nd^ Millennium BC, in a phenomenon that is usually connected with Mediterranean rather than Atlantic networks.

## Conclusions

The new evidence presented in this paper, brought together with information published previously, has allowed the most comprehensive review to date on the provision and exchange of amber in the Prehistory of Iberia. Several conclusions and priorities for future work can be highlighted:

- From a methodological perspective, thanks to the analyses carried out on both the core of vitreous amber and the oxidation layers of the objects from the tholos of Montelirio, Mound 74(XIII) from Los Millares and the artificial cave of Sao Paulo, we have been able to define the characteristic FTIR spectrum resulting from degraded Sicilian simetite. These spectra can be used as a reference to identify Sicilian amber even in highly deteriorated archaeological samples.
- The local origin of the amber from La Velilla emphasises the continued use of local resources from the Palaeolithic to the Bronze Age in the Northern region of Iberia, in stark contrast with the picture obtained from the South.
- We can verify that the arrival of Sicilian amber in the Iberian Peninsula started in the 4^th^ Millennium BC at least, and that it was likely integrated in broader Mediterranean exchange networks that intensified during the 3^rd^ Millennium BC, as attested to by amber finds as well as other exotic materials.
- After an apparent decline in the use of amber, Baltic succinite appears to replace Sicilian simetite in the Iberian Peninsula in the second half of the 2^nd^ Millennium BC, as evidenced by the analysis of Quinta do Marcelo and other sites; the more significant influx of Baltic amber will take place from the turn of the Millennium.

The new results thus fill some of the research gaps highlighted in our 2012 publication [9], while they extend the empirical base that allows us to infer the diachronic changes that took place in the amber exchange circuits. This panorama, however, leaves some unresolved issues that should be investigated in the future. One of them is the possible presence of amber in North African contexts from the 5^th^–3^rd^ Millennia BC and its archaeometric characterisation, not least considering the possible involvement of this region in the Mediterranean exchange network. Relatedly, a systematic exploration of North African amber resources should be warranted; there are currently no known geological deposits of amber in North Africa, except for a locality with amber in Tunisia that remains to be confirmed [91]. Another major priority in the research agenda is the need for further chronological precision to trace the introduction and spread of Baltic succinite in Iberia, the networks that made this possible, and the extent to which metals or other Iberian commodities were provided in return.

## Supporting information

S1 File Raw data of all samples analysed. (XLSX)
